# Quantification and Optimization of Standard-of-Care Therapy to Delay the Emergence of Resistant Bone Metastatic Prostate Cancer

**DOI:** 10.3390/cancers13040677

**Published:** 2021-02-08

**Authors:** Arturo Araujo, Leah M. Cook, Jeremy S. Frieling, Winston Tan, John A. Copland, Manish Kohli, Shilpa Gupta, Jasreman Dhillon, Julio Pow-Sang, Conor C. Lynch, David Basanta

**Affiliations:** 1 Integrated Mathematical Oncology Department, Moffitt Cancer Center and Research Institute, Tampa, FL 33612, USA; arturo@cancerevo.org; 2 School of Arts, University of Roehampton, London SW15 5PU, UK; 3 Department of Computer Science, University College London, London WC1E 6BT, UK; 4 Fred & Pamela Buffett Cancer Center, Department of Pathology and Microbiology, University of Nebraska Medical Center, Omaha, NE 68198, USA; leah.cook@unmc.edu; 5 Department of Tumor Biology, Moffitt Cancer Center and Research Institute, Tampa, FL 33612, USA; Jeremy.frieling@moffitt.org; 6 Department of Medical Oncology Mayo Clinic, Jacksonville, FL 32224, USA; tan.winston@mayo.edu; 7 Cancer Biology, Mayo Clinic, Jacksonville, FL 32224, USA; copland.john@mayo.edu; 8 Division of Medical Oncology, Huntsman Cancer Institute, University of Utah, Salt Lake City, UT 84122, USA; manish.kohli@hci.utah.edu; 9 Department of Hematology and Oncology, Taussig Cancer Institute, Cleveland Clinic, Cleveland, OH 44195, USA; guptas5@ccf.org; 10 Genitourinary Oncology Department, Moffitt Cancer Center and Research Institute, Tampa, FL 33612, USA; Jasreman.Dhillon@moffitt.org (J.D.); Julio.Powsang@Moffitt.org (J.P.-S.)

**Keywords:** computational model, mathematical oncology, androgen therapy resistance, bone metastatic prostate cancer, personalized treatment

## Abstract

**Simple Summary:**

Using a first-principles approach, we demonstrate how standard-of-care therapies for bone metastatic prostate cancer (BMPCa) patients can be optimized with the use of routine measurements to significantly delay the evolution of resistant disease, potentially extending overall patient survival.

**Abstract:**

Background: Bone metastatic prostate cancer (BMPCa), despite the initial responsiveness to androgen deprivation therapy (ADT), inevitably becomes resistant. Recent clinical trials with upfront treatment of ADT combined with chemotherapy or novel hormonal therapies (NHTs) have extended overall patient survival. These results indicate that there is significant potential for the optimization of standard-of-care therapies to delay the emergence of progressive metastatic disease. Methods: Here, we used data extracted from human bone metastatic biopsies pre- and post-abiraterone acetate/prednisone to generate a mathematical model of bone metastatic prostate cancer that can unravel the treatment impact on disease progression. Intra-tumor heterogeneity in regard to ADT and chemotherapy resistance was derived from biopsy data at a cellular level, permitting the model to track the dynamics of resistant phenotypes in response to treatment from biological first-principles without relying on data fitting. These cellular data were mathematically correlated with a clinical proxy for tumor burden, utilizing prostate-specific antigen (PSA) production as an example. Results: Using this correlation, our model recapitulated the individual patient response to applied treatments in a separate and independent cohort of patients (n = 24), and was able to estimate the initial resistance to the ADT of each patient. Combined with an intervention-decision algorithm informed by patient-specific prediction of initial resistance, we propose to optimize the sequence of treatments for each patient with the goal of delaying the evolution of resistant disease and limit cancer cell growth, offering evidence for an improvement against retrospective data. Conclusions: Our results show how minimal but widely available patient information can be used to model and track the progression of BMPCa in real time, offering a clinically relevant insight into the patient-specific evolutionary dynamics of the disease and suggesting new therapeutic options for intervention. Trial registration: NCT # 01953640. Funding: Funded by an NCI U01 (NCI) U01CA202958-01 and a Moffitt Team Science Award. CCL and DB were partly funded by an NCI PSON U01 (U01CA244101). AA was partly funded by a Department of Defense Prostate Cancer Research Program (W81XWH-15-1-0184) fellowship. LC was partly funded by a postdoctoral fellowship (PF-13-175-01-CSM) from the American Cancer Society.

## 1. Introduction

Approximately 30% of patients that are treated for primary prostate cancer will develop recurrent disease that presents either as biochemical recurrence (rising prostate-specific antigen (PSA) levels) or positive scans/biopsies of local or distant metastases [1,2]. The treatment options available to these patients, and those diagnosed with advanced metastatic prostate cancer, have typically involved initiation of androgen deprivation therapy (ADT) [3,4]. Although monitoring serum PSA levels remains a widely used indicator of tumor burden and therapy response, a better understanding and quantification of the unseen cellular evolution is needed [5,6]. First-generation anti-androgens such as Leuprolide (LHRH agonist) and Goserelin (GnRH agonist) are successful in slowing disease progression but, over time, resistance emerges despite the depletion of circulating androgens [7]. Surprisingly, the resistant cells that emerge are still dependent on androgens but have developed mechanisms to synthesize the hormone via increased activity of the enzyme CYP17A or the expression of splice variants of the androgen receptor that are ligand-independent/sensitive to very low androgen levels [8,9]. This has led to the development of novel hormonal treatments (NHTs), CYP17A inhibitors (abiraterone acetate), and highly specific androgen receptor antagonists (enzalutamide, apalutamide, darolutamide) [3,10,11,12]. Despite their success, the onset of castrate-resistant prostate cancer (CRPC) remains inevitable [13]. NHTs and chemotherapy (docetaxel/cabazitaxel) are effective in CRPC patients, but associated morbidities can limit dose and duration [14]. The upfront combination of ADT with chemotherapy or with NHTs is superior to ADT alone in extending the overall survival of patients diagnosed with recurrent hormone-sensitive metastatic prostate cancer [15,16,17,18]. More recently, the upfront intensification of therapy with the addition of abiraterone to ADT in the LATITUDE clinical trial showed improvement in overall survival [17]. This indicates that there is significant room to optimize the choice of standard-of-care therapies that are administered in hormone-sensitive and castrate-resistant prostate cancer states. However, doing so via clinical trials can be costly, time-consuming, and often does not take into account inter-patient heterogeneity. To redress this, computational and mathematical models derived from patient data that faithfully capture the biology of the disease can aid not only in predicting but also teasing out and quantifying the response to therapy, and ultimately, disease progression [19,20,21].

The key to the accuracy of mathematical models is the ability to measure tumor burden either by imaging or by using surrogate measurements [6,22]. While PSA has been widely used in mathematical models of intermittent and adaptive ADT [13,19,21,23,24,25,26,27], by focusing instead on the genomic heterogeneity of the underlying disease, it is possible to overcome many of the hurdles of the biomarker. For this, we generated a clinically oriented mathematical model of bone metastatic prostate cancer (BMPCa) without the need of data-fitting by quantifying empirical cellular data from analysis of human bone metastatic castration-resistant prostate cancer biopsies pre- and post-second-generation ADT (abiraterone, from Mayo clinic). The model results were mathematically correlated to a clinical biomarker, for which we used PSA as an example, and validated in a separate independent cohort of patient clinical records from Moffitt Cancer Center. Using only patient PSA values, the model recapitulated historical data on treatment response. Based on tumor response to treatment, the model was used to estimate the proportion of resistant sub-populations to first-generation ADT (such as LHRH and GnRH agonists) within each patient’s metastases. Consequently, resistance-informed algorithms were combined with the mathematical model to optimize how standard-of-care therapy is delivered to patients using PSA values as a surrogate for tumor response. Our approach suggests that, using existing therapeutics and basic clinical patient data with a mathematical understanding of disease evolution, we can delay the emergence of resistance, extend the efficacy of said therapies, and thus increase overall patient survival.

## 2. Methods and Materials

### 2.1. Immunofluorescent Staining and Analysis

To extract measurements of cellular behavior, it was decided to use cleaved Caspase-3 as a measure of apoptosis, and phosphorylated Histone-H3 as a proxy for division. Caspase-3 is known to be activated in apoptotic cells, while Histone H3 is specifically phosphorylated during both mitosis and meiosis. Both have been widely used as markers for cell death (both from intrinsic and extrinsic mechanisms) and cell division accordingly.

All patient information was obtained with appropriate IRB approval from the Moffitt Cancer Center (MCC-17402; n = 24). De-identified prostate cancer bone biopsies from patients experiencing ADT failure and before starting any new NHT for the castrate-resistant state (pre- and post-abiraterone acetate with prednisone treatment; n = 10) were provided by the Mayo Clinic, Rochester, MN. This was a sub cohort of patients enrolled into a larger biopsy-only trial performed at Mayo Clinic in castrate-resistant prostate cancer patients, (NCT # 01953640) the details of which have been previously published [28] and in which all patients provided a written informed consent. We selected patients with serial and successful biopsies of skeletal metastases for our study, which was not a stated aim of the larger study. Slides were dewaxed and rehydrated prior to transfer to antigen retrieval (1X Tris EDTA pH 9.0 in pressure cooker for 5 min followed by 20 min cooling to room temperature) and then blocked in 10% goat serum in 1X TBS for 1 h at room temperature. Antibodies (pan-cytokeratin, Sigma-Aldrich #C2562; phospho histone H3 Millipore #06-570; and cleaved caspase-3, Cell Signaling #9664S and matching IgG controls) were diluted (1:200) in blocking buffer and incubated overnight at 4 °C in a humidified chamber. After washing, slides were incubated in secondary antibodies (Alexaflour goat anti-rabbit 488, 568 Thermo Fisher Scientific, #A-32723 and #A-11011, respectively) at a 1:1000 dilution in blocking solution at room temperature under light-proof conditions for 1 h at room temperature prior to washing and aqueous mounting with Vectashield mounting medium containing DAPI (Vector Labs, # H-1200). At least three representative photomicrographs were taken for each sample and experimental condition. For semiquantitative analysis, regional images were segmented based on the intensity of staining using Definiens Tissue Studio Software, as we have previously described.

### 2.2. Model and Assumptions

The code is free to use (Apache License 2.0), written in Processing (processing.org), and can be downloaded at: github.com/d4r7hur/PSASim. The model was built around the National Comprehensive Cancer Network guidelines for the treatment of systemic castrate naïve prostate cancer, which suggest treatment with 1st-generation ADT (such as leuprolide and goserelin) or 2nd-generation ADT (enzalutamide or bicalutamide) either alone or in combination with chemotherapy (docetaxel or cabazitaxel) [29]. As the emergence of resistant disease is a constant in this patient cohort, we assumed that phenotypically, there are potentially up to 6 types of prostate cancer cell sub-populations that are either (1) naïve or can evolve to develop resistance to the following therapies: (2) 1st-generation ADT-resistant (ADT1^R^), (3) 2nd-generation ADT-resistant (ADT2^R^), (4) chemotherapy-resistant (CT^R^), (5) combination of ADT1^R^/CT^R^; or (6) combination of ADT2^R^/CT^R^.

**Figure 1:**
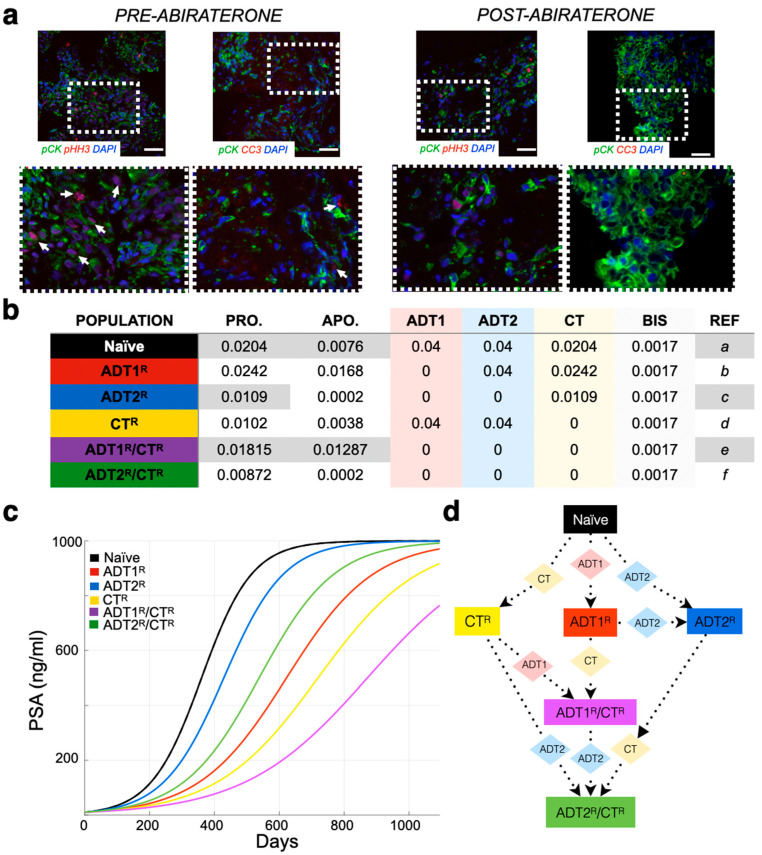
Parameterization of the mathematical model of recurrent prostate cancer. (**a**) Serial sections derived from bone metastatic prostate cancer patients (pre- and post-abiraterone) were stained for phospho-histone H3 (arrows, red) and cleaved caspase-3 (arrows, red) as readouts for proliferation and apoptosis, respectively, in pan-cytokeratin-positive (green) prostate cancer cells. Scale bars represent 100 μm. (**b**) Experimental and published parameters for prostate cancer growth rate per day pre- and post-treatment. Shown in the table are unitless rates of proliferation (PRO.), apoptosis (APO.), and the response (if any) to 1st-generation androgen deprivation therapy (ADT1) treatment (salmon), ADT2 treatment (light blue), chemotherapy (CT, light yellow), and palliative treatments (BISHP, light grey). Naïve population represents prostate cancer cells that are sensitive to all treatments, while subclonal populations have acquired resistance to one or more treatments. (**c**) These data were integrated into a mathematical model as daily logistic growth rates. Independent growth curves (i.e., each clone’s growth is individually simulated for comparison) are shown in ng/mL of prostate-specific antigen (PSA) (1 metastatic prostate cancer cell produces 9.8 × 10^−9^ ng/mL of PSA over a 24 h period). (**d**) Evolution of resistance (rectangles) to applied treatments (diamonds) show how the different clonal subpopulations emerge. We assume that bisphosphonates (BIS), radiotherapy (RT), and surgery (SURG) do not contribute to the development of resistance.

While tumor burden is calculated based solely on quantified cellular proliferation data (Figure 1a), a mathematical correlation can be made to any clinical biomarker, or sets thereof. As an example, we used PSA concentration as a clinical readout due to its wide use and availability. The literature reports that, on average, carcinomatous tissue contributes 3.5 ng/mL PSA per gram per day (with normal prostatic epithelium and benign hyperplastic tissue estimated at 0.1 and 0.3 ng/mL/g, respectively) [30]. A typical cell weighs 3.5 × 10^−9^ g [31]. As PSA and other factors make up approximately 20% of a cell’s weight, we estimated that 1 g of BMPCa tissue contains ~385,000,000 cells. Thus, 1 metastatic prostate cancer cell would produce 9.8 × 10^−9^ ng/mL of PSA over a 24 h period [32]. It has been further estimated that approximately 1 × 10^11^ cells make up the total cellular volume of bone marrow in adult humans [33]. Transforming this number into the upper-limit scenario where the bone marrow is made up entirely of cancer cells, we determine that the maximum theoretical carrying capacity ($C_{cap}$) for tumor growth is when the PSA levels reach 1000 ng/mL (~1.08 × 10^11^ cells) (Figure 1). This value was also used as a conjectured clinical endpoint. A caveat of this proxy is that, in the late stages of the disease, less luminal tumor cells might evolve to produce less PSA per cell or even stop producing it all together. While a quantification of this antigen production differential is outside of the scope of this research, any other biomarker or set thereof (such as immune cell response measurements) can be correlated with the model.

**Figure 2:**
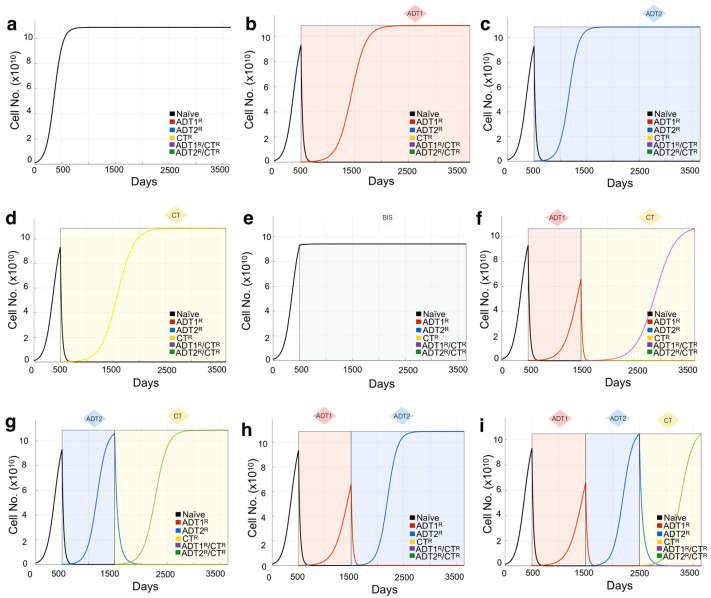
Mathematical model of growth kinetics for naïve prostate cancer clones and the effect of applied therapies on evolution. The simulations are initialized with an initial 10 PSA (1.08 × 10^9^ cells) made up entirely of naïve cells. (**a**) Cancer grows in the absence of treatment until reaching the maximum carrying capacity (1000 PSA ~ 1.08 × 10^11^ cells). (**b**–**e**) The effects of individual treatments (a continuous application from day 500 for demonstration purposes) on the clonal composition of the tumor over time. Resistant clones evolve from the initial naïve population and now compete for space within the carrying capacity of the tumor. Shading represents treatment. (**f**–**i**) The sequence of treatment application has profound effects on the evolution of the resistant subclones. Treatment is given in 1000 day intervals just for demonstration purposes.

When interpreting the patient’s clinical data, we consider (1) that the initial PSA value could represent hormone-sensitive recurrent cancer (Stage M0) and, therefore, could be a homogeneous population of naïve prostate cancer cells (no resistant sub-populations present). While this is an important first step in determining theoretical initial conditions that can evolve freely in response to treatments, a heterogeneous population could give rise to the same PSA. To tackle this, we also simulate different initial populations whose evolution might recapitulate the data better. We consider that an initial resistance to 1^st^-generation ADT could be present and simulate key scenarios ranging from an initial presence of 10% to 100%-resistant populations. In this coarse-grain approach, we will not consider any other initial resistance to reduce complexity in the analysis of the results. We assume that (2) the treatments as reported in the patient’s history were applied in duration and intensity according to the standard-of-care therapy [29], and (3) treatments such as bisphosphonates, surgery, and radiation were applied as palliative treatments, i.e., these treatments decrease PSA by debulking the tumor but do not impact tumor heterogeneity and do not generate resistance (Figure 2) (Figure S2). While our previous work shows that the effects of bisphosphonates are known to have antitumor effects, the impact they might have on generating subpopulations of cells is currently unknown [34].

The units used in the mathematical model are days for time and cell number for growth. For each simulated day, a set of equations that use our quantification of the daily proliferation of the different kind of cells (Figure 1b) is calculated. Each equation calculates the cell population growth for the next day ($T_{day+1}$) by adding to the current tumor cell population ($T_{day}$) its own logistic growth. The growth rate described by the logistic equation is reduced as the total population of cells (the sum of all cell subpopulations, $T_{total}$) approaches the maximum carrying capacity ($C_{cap}$ ~ 1.08 × 10^11^ cells). The growth rate differences between the cell populations are based on their resistance-specific proliferation (p) and apoptotic (a) rates calculated as a percentage chance in cell number per day for every cell kind (Figure 1b). More generally:

(1) $$T_{day+1}=T_{day}+\left(1-\frac{Ttotal}{Ccap}\right)\left(p-a\right)*T_{day}\;$$

This is then calculated for each cancer cell population and their intrinsically different growth rates. In the absence of treatments, the different cell kinds are shown growing independently (Figure 1c), or in direct competition in (Figure S2).

The model can accept clinical readouts such as PSA as a starting point for the simulation. Any biomarker can be correlated to the estimated number of cells (tumor burden) that would produce such an amount. For example, if the patient presents a PSA of 10 ng/mL at day 1, considering initially that they are all naïve cells, we calculate that the total number of naïve cancer cells at that time is 10/9.8 × 10^−9^ = 1.02041 × 10^9^ cells. Our data measured the daily proliferation (p) and apoptotic (a) rates of naïve cancer cells as 0.0204% and 0.0076%, respectively (Figure 1b). Therefore, to calculate the total number of naïve cells for day 2, we add to the total number of naïve cells from day 1 a logistic-modulated net growth of [1.02041 × 10^9^ cells × (0.0204 − 0.0076) × 0.99] = 1.29306 × 10^7^ new cells. Therefore, we have gained ~0.1 ng/mL of PSA for a total of ~10.1 ng/mL on day 2, as in this example, there are no other tumor clones (Figure 2a).

Treatment response was parametrized from the best available literature (Figure 1b). Chemotherapy rates are based on the effectiveness of the treatment being proportional to division attempts [35]. Concurrent application of treatments was not considered for this study, as our aim was to first understand the linear effects of single treatments. A treatment (*Hx*) is applied; it has a direct impact only on the cells that are sensitive to it. This is calculated daily (or only once in the case of surgery and radiation) after calculating growth, using the rates shown in Figure 1b as:

(2) $$T_{day+1}=T_{day}-\left(Hx*T_{day}\right)$$

To model resistance, we consider that a small proportion of cells that survive treatments have acquired key mutations that enable them to counteract the intervention. It has been calculated that 0.00005% is the probability of a random mutation on key tumor-associated genes per cell per day [36], which we have used as the percentage of cells that become resistant every day to treatment. For instance, an initial population of treatment-naïve cells is sensitive to every treatment. Upon therapy administration, this naïve population is reduced through apoptosis with the rates described in Figure 1b, with 0.00005% being able to mutate into a subpopulation resistant to the treatment being applied. Each new resistant subpopulation acquires new proliferative and apoptotic rates, an evolutionary tradeoff for the advantage gained. Responses to 1st- and 2nd-generation ADT are based on our experimental observations and the literature [37,38]. For chemotherapy treatment, we model resistance as a cost resulting in a growth penalty compared to naïve cells [35].

As the majority of recurrent prostate cancers are sensitive to 1st- or 2nd-generation ADT, we assume the initial phenotypic clonal composition of the BMPCa to be either ADT-naïve, or partially ADT1^R^-resistant. To estimate the initial heterogeneity of the tumor, 11 simulations with different initial resistance ratios are generated: 100% naïve; a mixed ratio of naïve and ADT1^R^ populations in 10% increments (90% Naïve: 10% ADT1^R^, 80% Naïve: 20% ADT1^R^, etc.); or 100% ADT1^R^. We measure the Euclidean distance between the predicted growth curves and the patient PSA history to select the scenario that is closer to the data, shedding light on the initial heterogeneity at the time of presentation. The resistant types other than ADT1^R^ are not considered for determining the initial heterogeneity, but can subsequently emerge when therapies are applied (Figure 2).

### 2.3. Resistance-Informed Intervention-Decision Algorithm

Once the initial heterogeneity has been estimated from the simulations, we aim to exploit this knowledge to design a patient-specific application of the current standard of care. We created an intervention-decision algorithm that utilizes the information on the theoretical heterogeneity of each patient at presentation and chooses a sequence of treatments for each case. Each treatment is applied for 28 days (3 weeks on, 1 week holiday). Key cases for the algorithm to choose from were designed in close collaboration with clinicians, making sure the suggested sequence does not conflict with that of the current standard of care. The algorithm is as follows: If the number of cells in the simulation exceed 1.02 × 10^8^ cells (1 ng/mL of PSA) and no treatment is currently being applied, the algorithm selects the single treatment that would impact the largest sub-population of cells (Figure 1d shows the susceptibility of the populations to treatments). The algorithm can select from these 4 clinically plausible cases: (Case 1) If naïve is the largest population, the model will select ADT1. (Case 2) If ADT1R is the largest population, the model applies ADT2. (Case 3) If it is resistant to all ADTs, then CT will be applied. (Case 4) If the largest clone has become resistant to both ADTs and CT, palliative care (bisphosphonates) will be applied. At the end of each treatment cycle (28 days), the algorithm starts over.

### 2.4. Model Validation and Limitations

Based on sensitivity and resistance to 1st- and/or 2nd-generation ADT and/or chemotherapy, we deduced the existence of 6 possible populations (naïve, ADT1^R^, ADT2^R^, CT^R^, ADT1^R^/CT^R^, ADT2^R^/CT^R^, described in Model and Assumptions). The growth rates for these populations distilled from the literature or experiment were then integrated into logistic equations driving the mathematical model (Figure 1b). We simulated the growth of each population individually seeded into the model in isolation (plots presented together for illustrative purposes) and in treatment-free conditions (Figure 1c). Each clone was found to grow exponentially until they reach the carrying capacity of the system, 1000 ng/mL of PSA or the equivalent of 10^11^ cells (Figure 1c). If the clones are initially found together, sharing the same carrying capacity, cells compete for resources with the naïve population having the advantage in proliferation (Figure S2a). Depending on the standard-of-care treatment applied to the system, one of five populations can arise provided the treatment is given in a sustained and continued fashion (Figure 1d). Treatment of the naïve population with ADT1 gives rise to the emergence of an ADT1^R^ population, and treatment of the ADT1^R^ population with ADT2 or CT gives rise to an ADT2^R^ or ADT1^R^/CT^R^ population, respectively, as clinically expected (Figure 2).

To validate the expected treatment dynamics under ideal conditions and explore the limits of the mathematical model, we focus on the case of a tumor initially made up of 100% naïve cells. To set a baseline, we simulate an initial PSA of 10 ng/mL and let the cells grow until the carrying capacity of the system in the absence of any intervention is naturally reached (Figure 2a). Applying 1st- or 2nd-generation ADT or chemotherapy reduces the naïve population but promotes the emergence of populations resistant to each of those therapies over time (Figure 2b–d). Patients with BMPCa are often administered bisphosphonates that, in clinical trials, delayed time to pathological fracture but did not improve the overall survival of patients with CRPC [39]. We therefore modeled the effect of bisphosphonates on cancer cell growth as purely palliative and that the effect would be equal across the clonal populations, generating no further resistance. Simulations show that, in the event of bisphosphonate treatment, the growth of all clones is impacted, but the naïve prostate cancer cell population continues to grow toward the carrying capacity of the system (Figure 2e). Finally, we demonstrate how the sequence of treatments has a profound effect not only on the reduction in the total number of cells, but also on the emergence of resistant phenotypes (Figure 2f–i). This experiment was also performed using a tumor with a heterogeneous population, showing how initial conditions affect the response to treatment (Figure S2). Having shown the confidence and limitations of this theoretical model, parametrized by experimental data and best available literature, we sought to recapitulate the clinical histories and tease out the effects of treatments in real patients from a completely independent cohort.

## 3. Results

### 3.1. Defining the Impact of ADT on Prostate Cancer Growth Rates

Previous studies have examined the effects of 1st-generation ADT and chemotherapy on the growth of prostate cancer [35,37,38]. Novel to this work, the growth rates of prostate cancer cells in bone subsequent to treatment with 2nd-generation ADT were determined. We examined the proliferative and apoptotic indices in serial human BMPCa biopsies pre- and post-abiraterone treatment (Figure 1a and Figure S1). Our extracted data demonstrate that the proliferation index of pan-cytokeratin-positive prostate cancer epithelial cells in the pre-treatment group was 2.42%, while in the post-treatment group, it was reduced to 1.09% using phospho-histone-H3 as a readout (Figure 1b). Cleaved caspase-3 staining revealed an apoptotic index of 1.68% in the pre-treatment group, while the post-treatment index was 0.02%. This parametric description of the data is consistent with previous studies examining the effects of 1st-generation ADT on prostate cancer growth in human specimens and in preclinical animal models (Figure 1b) [37,38].

### 3.2. Modeling Individual Patient Responses to Standard-of-Care Therapy

**Figure 3:**
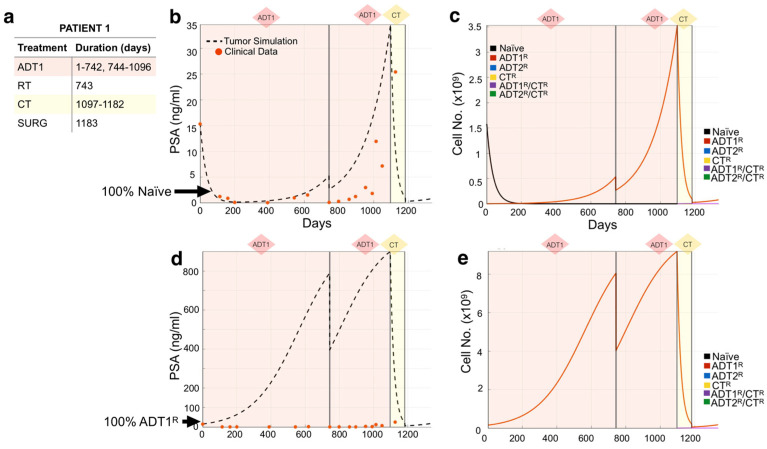
Tracking individual patient response to treatment. (**a**,**b**) Patient 1’s treatment information consisting of the time intervals for the application of 1st-generation androgen deprivation therapy (ADT1), radiotherapy (RT), chemotherapy (CT), and surgery (SURG) was obtained (**a**) and applied to a simulation seeded with 100% naïve prostate cancer cells (**b**). Red dots indicate the patient’s PSA values over time. Dotted lines indicate the total tumor burden simulated by the mathematical model. (**c**) Analysis of cancer cell evolution in silico over time. (**d**,**e**) Altering the initial assumption of resistance naivety, a simulation that starts with 100% AR1-resistant cells fails to recapture the data (note the increase of an order of magnitude in the theoretical level of PSA, compared to data). This analysis suggests that the patient presented with an initial population of 100% naïve and 0% AR1 resistance. Resistance likely emerged after treatment was applied.

We used the mathematical model to recapitulate the response of individual patients to standard-of-care treatment from an independent Moffitt Cancer Center cohort (n = 24). Following our methodology, we used each patient’s medical record information (Figure 3a) to initialize and simulate their actual treatment regimen (days under treatment) in the mathematical model. We then explored different initial heterogeneity scenarios of the tumor over time, and their response to the treatment regime (Figure 3c), decomposing the clinical PSA into the individual contributions from the different cell subpopulations, a unique aspect of this modeling approach. Comparing the model outputs with the clinical PSA data points over time, we observed that the simulation initialized with 100% naïve cells recapitulated the course of Patient 1’s disease best (Figure 3b). The simulation revealed that the application of 1st-generation ADT (ADT1) probably led to the quick emergence of the ADT1^R^ population, accounting for the rise in PSA despite the continuous application of treatment. Surgery to debulk the tumor and chemotherapy significantly impacted the disease burden at day 1183, but by day 1335, the patient was recorded as deceased. To investigate the role that resistance has on the evolution of the disease, we show that simulating an initial population of 100% ADT1^R^ (no naïve cells) under the same treatment regime would not recapitulate the clinical outcome (Figure 3d,e). The model therefore suggests that the majority of the patient’s disease was initially sensitive to ADT1 treatment, but quickly developed complete resistance.

**Figure 4:**
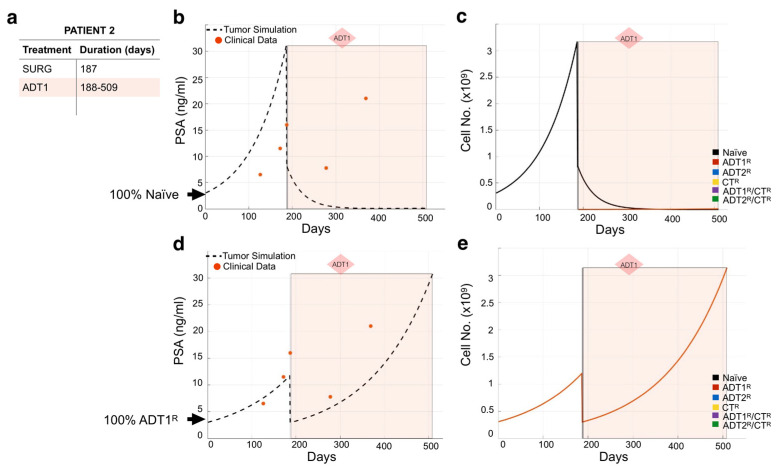
Assessing dominant cancer cell population at the time of presentation using the mathematical model. (**a**,**b**) Patient 2’s treatment information was obtained (**a**) and applied to a simulation seeded with 100% naïve prostate cancer cells (**b**). Red dots indicate the patient’s PSA values over time. Dotted line indicates the total tumor burden simulated by the mathematical model. (**c**) Analysis of cancer cell evolution over time in response to applied therapy. (**d**) Reinitialization of Patient 3’s simulation with 100% ADT1^R^. (**e**) Analysis of cancer cell evolution in silico over time subsequent to initialization with 100% ADT1^R^ population. These data suggest that the patient presented with an initial population of 100% AR1 resistance.

We used the same methodology to model the remaining 23 patients (Figures S3–S25). As expected, not all individual patient simulations that start with the assumption of a 100% naïve population of cancer cells recapitulated the course of their disease. For example, simulations for Patient 2 show that 100% naivety has no correlation between theoretical tumor burden and real PSA readings over time in response to the patient’s treatment regimen (Figure 4a,b). If this had been the case, the application of 1st-generation ADT (ADT1) would have eliminated the naïve cancer cell population (Figure 4c). This indicates that the patient may have presented with cancer already resistant to ADT1. By simulating the different scenarios for initial conditions, we found that considering that the patient presented with disease that was 100% ADT1-resistant (ADT1^R^) remarkably captures the patient’s response. With this seemingly simple change in the initial conditions, the tumor burden simulation now closely aligned with the PSA values recorded from the patient over time (Figure 4d), and allows for a deeper understanding of the impact of the treatment regime. Assessing the theoretical tumor heterogeneity shows the dominance of the ADT1^R^ population throughout the patient’s treatment course with the exception of the applied surgery at day 187 (post-presentation) that resulted in the significant reduction in the ADT1^R^ population burden (Figure 4e). Retrospectively, the simulation (based on the first 2–3 PSA readings) suggests that the patient’s cancer was already predominantly ADT1^R^, not responding to the treatment applied. In a clinical setting, this kind of time-sensitive information could be leveraged in a similar fashion to make informed decisions on the treatment to use.

### 3.3. Design of Resistance-Informed Patient Treatment Strategy to Extend Overall Survival

**Figure 5:**
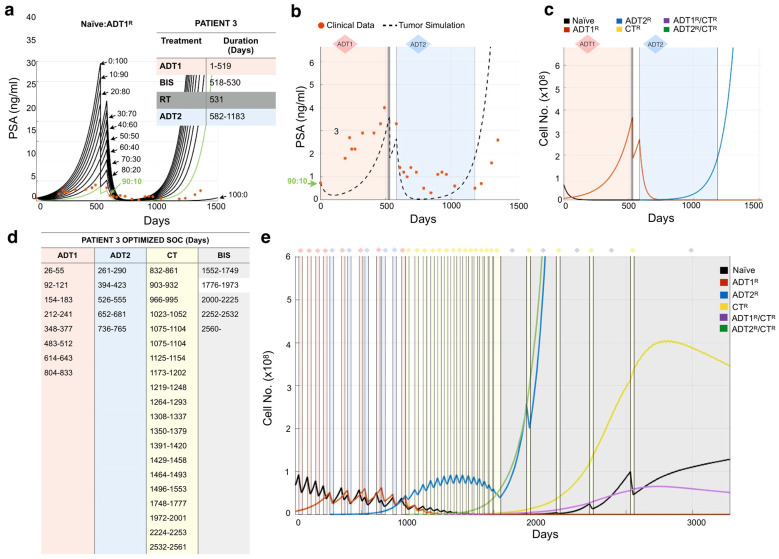
Determining tumor heterogeneity at the time of presentation. (**a**) Patient 3’s treatment information was obtained (inset) and applied to the mathematical model seeded with varying ratios of naïve:ADT1^R^ prostate cancer cells. Naïve: ADT1^R^ (80:10, green) was identified as the most accurate representation. (**b**) The mathematical model was initialized with an 80:10 (Naïve: ADT1^R^) population and the tumor burden was compared to recorded PSA values over time (red dots). (**c**) Analysis of cancer cell evolution over time in response to applied therapy. (**d**,**e**) Personalized treatment regimen developed for Patient 3 (**d**) and the impact on cancer cell evolution over time. (**e**) Periods of treatment are represented by shaded bars.

A major advantage to this mathematical modeling approach is that standard-of-care treatments can be optimized with little clinical data to delay the emergence of resistant disease. Following our established methodology for Patient 3, we initially considered that the prostate cancer was composed of 100% Naïve cells, 100% ADT1^R^ cells, or a blend of treatment-naïve and ADT1^R^-resistant clones (Figure 5a). Simulating adjustments of 10% in the ratio for each initial population, we found that a blend of 90:10% Naïve:ADT1^R^ populations provided simulations that closely matched the patient’s PSA values and their response to applied therapies (Figure 5a,b). Analysis of cancer evolution in this patient showed that the naïve population quickly gave way (within 50 days) to the growth of the ADT1^R^ population during treatment with ADT1. Subsequent treatment of the patient with radiotherapy and application of ADT2 successfully reduced ADT1^R^ numbers. However, from day 600 onward, there was an emergence of ADT2^R^ cells that continued until the endpoint (Figure 5c). Knowing a priori the initial resistance to ADT1 treatment can then be utilized to optimize how standard-of-care therapy could be delivered.

After successfully recapitulating the patient’s PSA data, we applied a Resistance-Informed Intervention-Decision Algorithm (described in Materials and Methods), informed by the knowledge of Patient 3’s estimated initial resistance (90:10% Naïve: ADT1^R^). For Patient 3, the optimal treatment sequence designed suggests that early ADT1 cycles work best, followed by a transition to ADT2 at day 261 post-patient presentation. Alternate cycles of ADT1 and ADT2, combined with periods of no treatment so as to allow for the rebound of naïve populations, keep the tumor in check until day 1800. Alternate cycles between CT and bisphosphonates prolong patient survival until day 2200 when the PSA levels reach those at which the patient was recorded as being deceased (Figure 5e,f). Taken together, the optimized treatment regimen designed by the mathematical model extended the theoretical overall survival of the patient from 1523 days to over 2240, an almost 2 year increase. We used this methodology to simulate and assess all the patients in our independent Moffitt cohort (n = 24), obtain their theoretical initial heterogeneity, and further calculate a personalized treatment strategy for each (Figures S3–S25).

## 4. Discussion

Based on recent clinical trial results, there is clearly room for optimizing the effectiveness of standard-of-care therapies used for the treatment of BMPCa. Here, we have shown that a mathematical model, integrating experimental parameters and clinical data, can forecast the course of a patient’s disease. Importantly, it also uniquely provides new insight as to the underlying heterogeneity of the recurrent cancer at the time of presentation. The evolution of the cancer in the context of applied ADT or chemotherapies can also be tracked and analyzed. Finally, using the information of the initial resistance in a treatment design algorithm, we also provide a methodology on how treatment regimens could be improved and personalized for each patient so as to limit the emergence of ADT/chemotherapy-resistant disease, thereby extending overall survival. The theoretical gains observed for every patient are feasible in light of recent clinical observations with evolutionary-enlightened treatments for castrate-resistant prostate cancer [21,40]. Moving forward, we expect that acquiring the initial (2–3) PSA measurements pre- and post-treatment would allow the mathematical model to predict-patient specific outcomes. For example, a patient would present with rising PSA levels indicative of recurrent disease and be prescribed ADT1. The response of PSA (2–3 measurements) subsequent to the first cycle would allow for model calibration to predict the underlying heterogeneity and time to the emergence of resistant disease. An optimized sequence of treatments could then be proposed for each patient at that juncture and provide a useful guide for the medical oncologist.

We modeled one-month cycles to ensure clinical feasibility, but it is plausible that patients who have adverse reactions (to chemotherapy, for example) may not be able to complete the proposed cycle. In this scenario, additional constraints could be imposed in collaboration with medical oncologists to restrict the application of chemotherapy. While the predicted overall survival may be less than the unconstrained algorithm, the model should be able to maximize the efficacy of applied therapy and improve overall patient survival. Notably, the model did not consider the possibility of combining therapies, as modeling collateral sensitivity and toxicity would require available clinical and pre-clinical [41]. This will be considered in future studies as new information emerges. Furthermore, this model did not integrate any information on putative genomic drivers in hormone-sensitive and castrate-resistant states that have been associated with poor prognosis [42]. While falling outside of the scope of this project, such information would be useful to capture the variability of drug–host interactions and help modulate not only the duration but the intensity of the standard of care to offer the patient a better quality of life.

The idea of using PSA levels to extend the usefulness of applied ADTs is not without precedent. Several studies have examined the effect of applying ADT intermittently or have used PSA levels to apply ADT in an adaptive manner [21,43,44,45]. In the latter approach, several clinical trials are also underway (NCT03674814, NCT03418324, NCT03246347, NCT0336072, for example). A caveat is the heavy reliance on PSA information derived from the patient to guide the models. While it is widely available and there is good reason to believe that PSA for monitoring the therapy response is useful in most cases, PSA is only one of the 200+ androgen response elements (AREs) that could be used as a proxy for tumor burden. Indeed, although correlating well with tumor burden, there is no study that has shown that PSA levels in themself can predict treatment failure. As such, there may be better predictive biomarkers that predict responses. Research is currently being done on the elucidation of microenvironmental and immune biomarkers that could be routinely extracted and analyzed with Artificial Intelligence (AI) methodologies for better diagnosis [46]. In our data, we observed that, although the trend in terms of correlation to tumor growth and response to treatment was similar, PSA levels amongst the patient cohort examined (n = 24) varied greatly. Using PSA as a readout for tumor growth can also be risky as there is evidence that in some patients, PSA levels are not reflective of the tumor burden. Here, the decrease in PSA levels is used only as a proxy to transform patient data for use with our biologically derived model. As the framework is derived from in-house accurate cellular measurements and accepted biological parameters, any other clinical biomarker or sets thereof can therefore be linked to the model to guide therapy decision-making.

PSA has been widely used as a correlative for tumor burden. However, many new less-intrusive markers are being developed. The mathematical model captures different cell population dynamics and is not dependent on PSA. As the clinical data measure PSA, a conversion is made so that a comparison between model prediction and data can be established. As other biomarkers emerge, and as clinicians start making use of them, our workflow will be able to take advantage of those developments and replace/complement PSA with more accurate proxies. We envision that more routinely extracted biomarkers that correlate through AI techniques to tumor burden (such as a mix of immune cell population, free PSA, and other novel markers) will incentivize patients to follow up with their progression tracking. Furthermore, we hope that a demonstrative version of this model could be used to help patients understand their individual disease and the importance of tracking their progression.

While the focus of this work is to investigate the resistance of treatments due to therapeutic pressure, work is yet to be done on integrating other known population risks. Factors such as age, smoking, race, diet, geographical location, and overall wellbeing play a key role in cancer progression and treatment response. As we are able to pin down the biological rules that drive cancer evolution through treatments, it will be possible to incorporate this important information and study its role on treatment, prognosis, and quality of life.

## 5. Conclusions

In this work, we have distilled and quantified basic cellular parameters previously not reported in the literature to increase our understanding of the basic biology underpinning resistance. Leveraging this, and in close collaboration between clinical oncologists, experimental biologists, and mathematical modelers, we generated a general mathematical framework to study the onset of resistance to key standard-of-care therapies in the context of tumor evolution in BMPCa. Once this model was validated with an independent cohort of patient histories, we were able to estimate details about heterogeneity upon presentation for each patient. We used the model to track the dynamics of resistant phenotypes in response to treatment. A key aspect is that we worked from biological first-principles without resorting to data fitting, thus bridging cellular biology and clinical studies. Finally, we propose to use this approach to design new patient-specific treatment sequences to delay the onset of resistance. The core idea is that even basic and imperfect clinical information can be leveraged mathematically to help decide when to apply or withdraw ADT so as to not completely ablate the treatment-naïve population, delay resistance by exploiting intra-tumor competition, and extend ADT effectiveness. In this case, we have used PSA as a proxy for tumor burden due to its wide availability even if, as a biomarker, it is an imperfect one. Our approach can incorporate any other such biomarker or combination of them. The model presented herein represents a significant advance in that it tackles the question of heterogeneity and the evolution of resistance in BMPCa.

The model assesses clonal resistance and enables the application of novel algorithms to prevent the emergence of resistant disease, thereby extending the longevity of effective standard-of-care therapeutics. Future work will focus on modeling the relationship between additional markers described in the literature, such as immune cell response in place of PSA, thus improving the ability of the mathematical model to predict patient outcomes [47]. Our resistance-informed treatment design for each patient demonstrates how single-agent therapies can be applied in a cyclical manner within the guidelines of the current standard of care. In conclusion, we have shown that integrating basic molecular and cellular data into a bottom-up, patient-specific mathematical model can be used to recapitulate, understand, and potentially improve clinical outcomes for prostate cancer patients.

## Data Availability

The data presented in this study are available in this article (and supplementary material).

## Supplementary Materials

The following are available online at https://www.mdpi.com/2072-6694/13/4/677/s1, Figure S1: Representative IgG controls for phosphohistone H3 (pHH3, red, a) and cleaved caspase-3 (CC3, red, b), Figure S2: Evolution of equally distributed naïve and resistant phenotypes to applied treatments, Figures S3–S25: De-identified patient simulations and patient-specific treatment optimization.
