# Supplementary material for: Quantification and Optimization of Standard-of-Care Therapy to Delay the Emergence of Resistant Bone Metastatic Prostate Cancer
Source: Cancers (Basel). 2021 Feb 8;13(4):677. doi: 10.3390/cancers13040677 (PMC7915310; doi:10.3390/cancers13040677)
Supplement: Supplementary file 1 [file cancers-13-00677-s001.pdf]

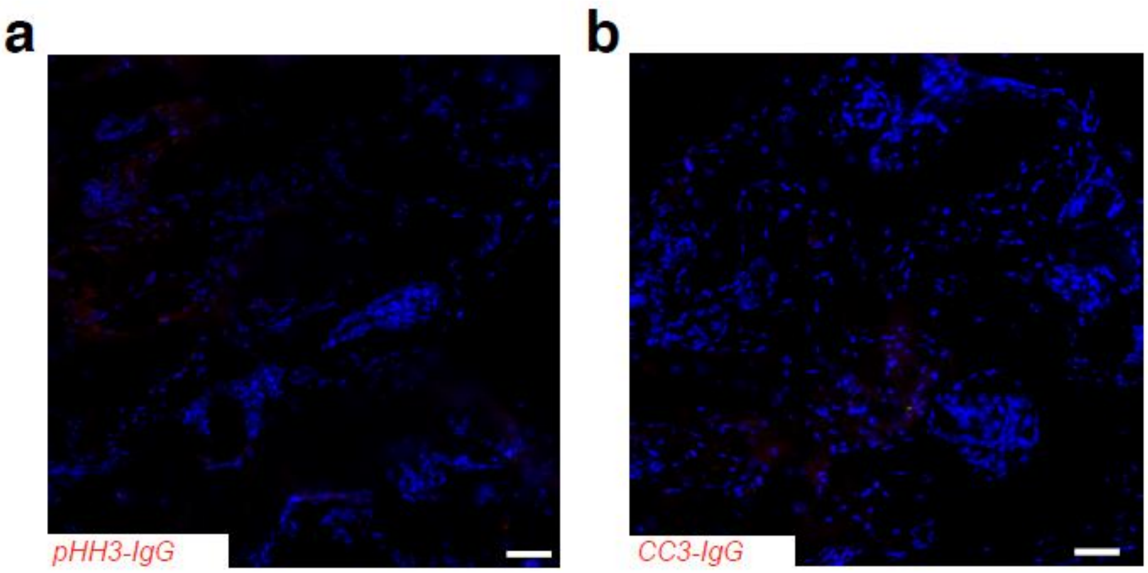

Figure S1. Representative IgG controls for phospho-histone H3 (pHH3, red, a) and cleaved caspase-3 (CC3, red, b). DAPI (blue) was used to stain nuclei. Scale bars represent 100 m.

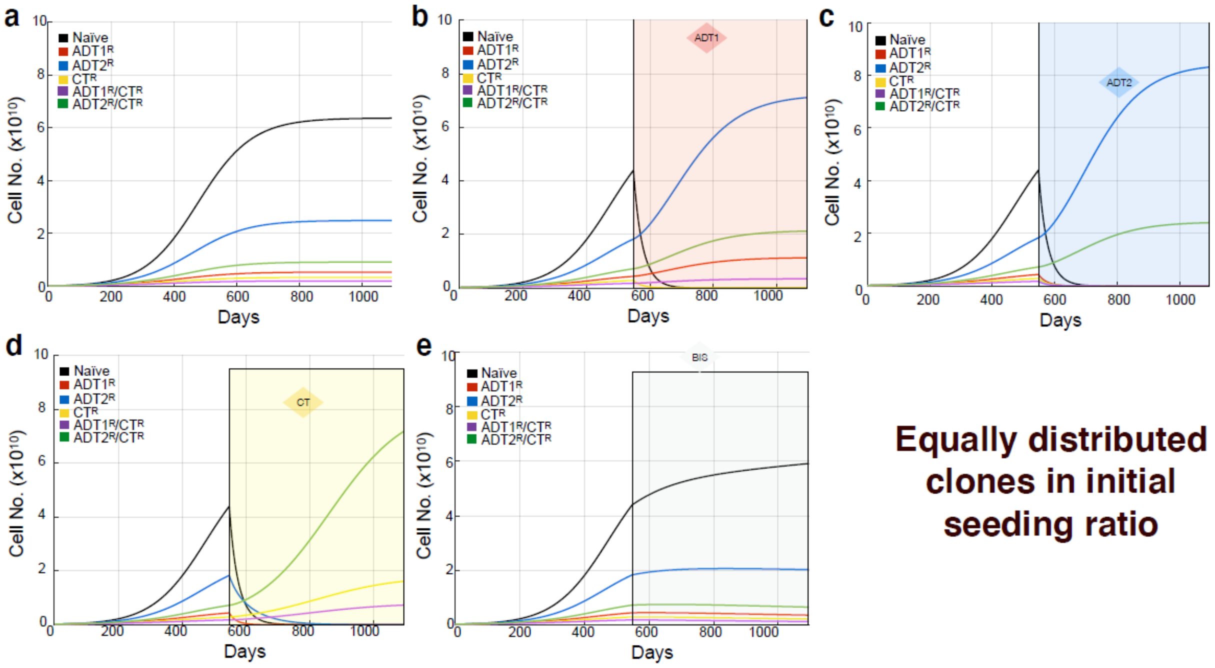

Figure S2. Evolution of equally distributed naïve and resistant phenotypes to applied treatments. The mathematical model of growth kinetics show naïve and resistant prostate cancer clones evolving differently than previously shown homogeneous initial conditions (Figure 2). These simulations are initialized with an initial 10 PSA (1.08 × 10^9 cells) distributed equally across the 6 clone subpopulations: naïve, ADT1R, ADT2R, CTR, ADT1R/CTR and ADT2R/CTR. **a**, Cancer grows in the absence of treatment until reaching the maximum carrying capacity (1000 PSA ~1.08 × 10^{11} cells), showing competition amongst cells for resources. In the absence of treatment, naïve cells have a growth advantage. **b**–**e**, The effects of individual treatments (continuous application) on the clonal composition of the tumor over time. Bisphosphonates can be applied continuously, but radiation and surgery can only be used once to debulk the tumor.

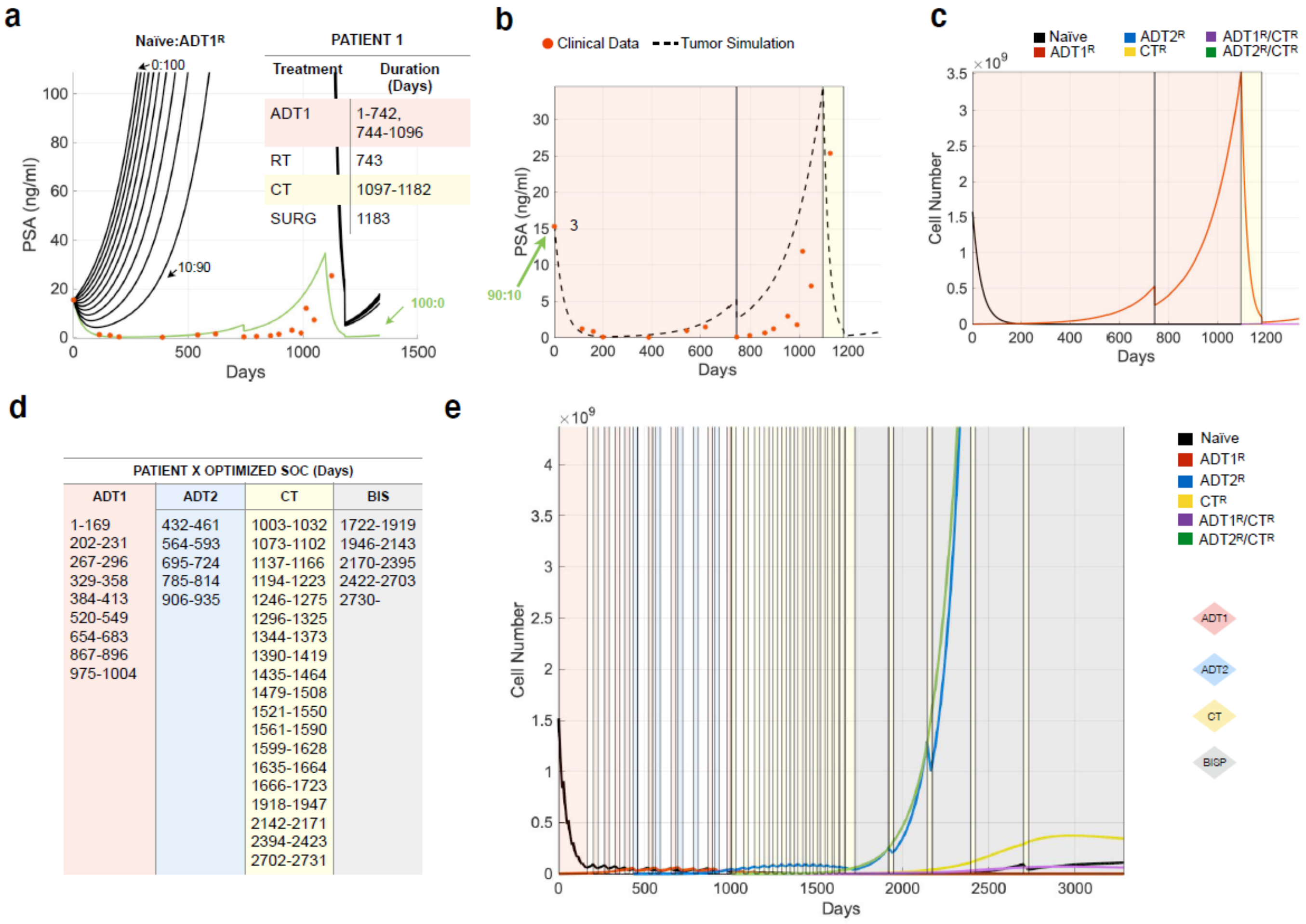

PATIENT X OPTIMIZED SOC (Days)

ADT1	ADT2	CT	BIS
1-169	432-461	1003-1032	1722-1919
202-231	564-593	1073-1102	1946-2143
267-296	695-724	1137-1166	2170-2395
329-358	785-814	1194-1223	2422-2703
384-413	906-935	1246-1275	2730-
520-549		1296-1325	
654-683		1344-1373	
867-896		1390-1419	
975-1004		1435-1464	
		1479-1508	
		1521-1550	
		1561-1590	
		1599-1628	
		1635-1664	
		1666-1723	
		1918-1947	
		2142-2171	
		2394-2423	
		2702-2731	

Figure S3–S25. De-identified patient simulations and patient-specific treatment optimization.

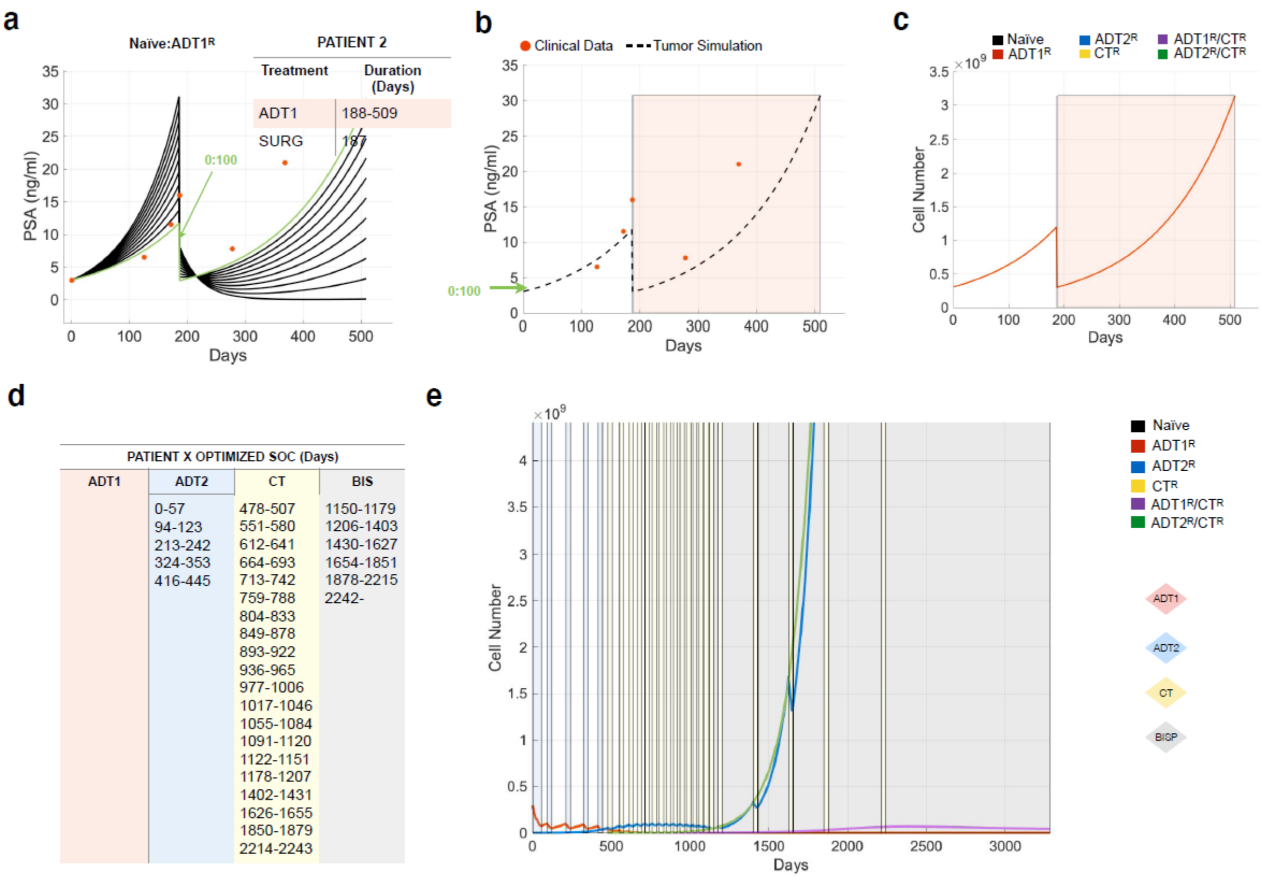

Figure S4. Araujo et al.

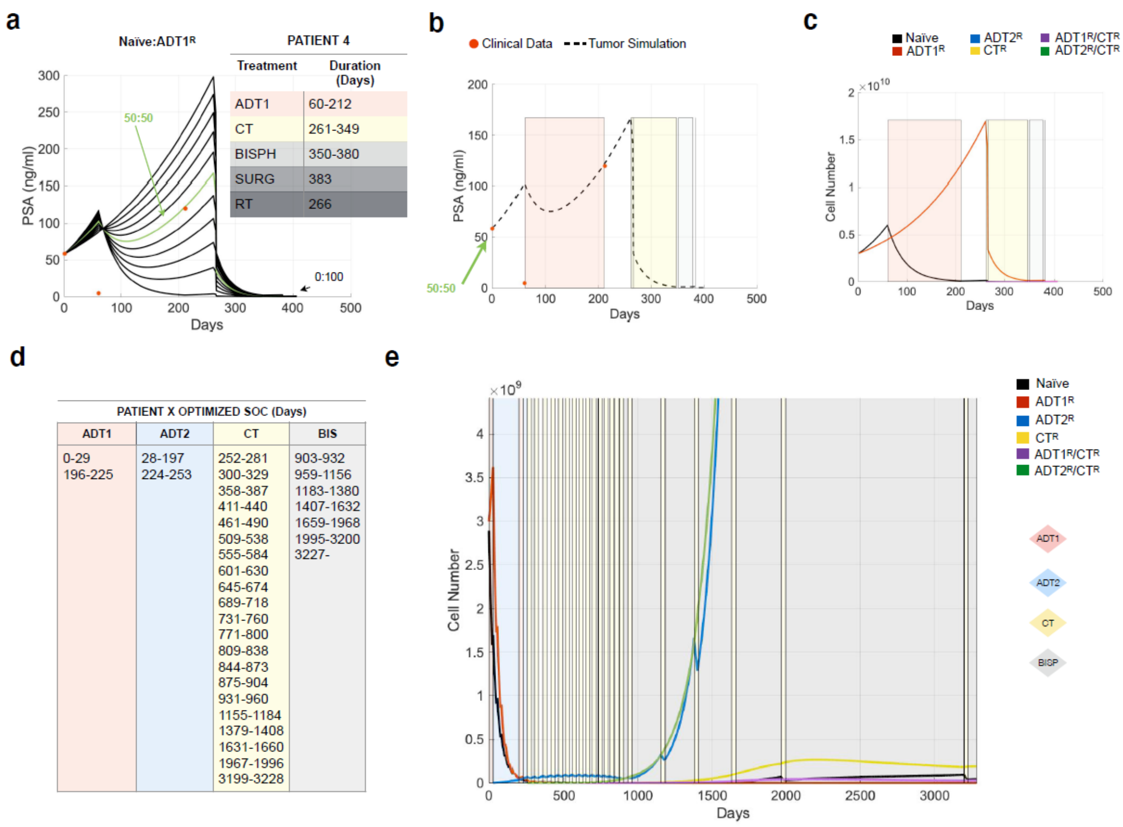

Figure S5. Araujo et al.

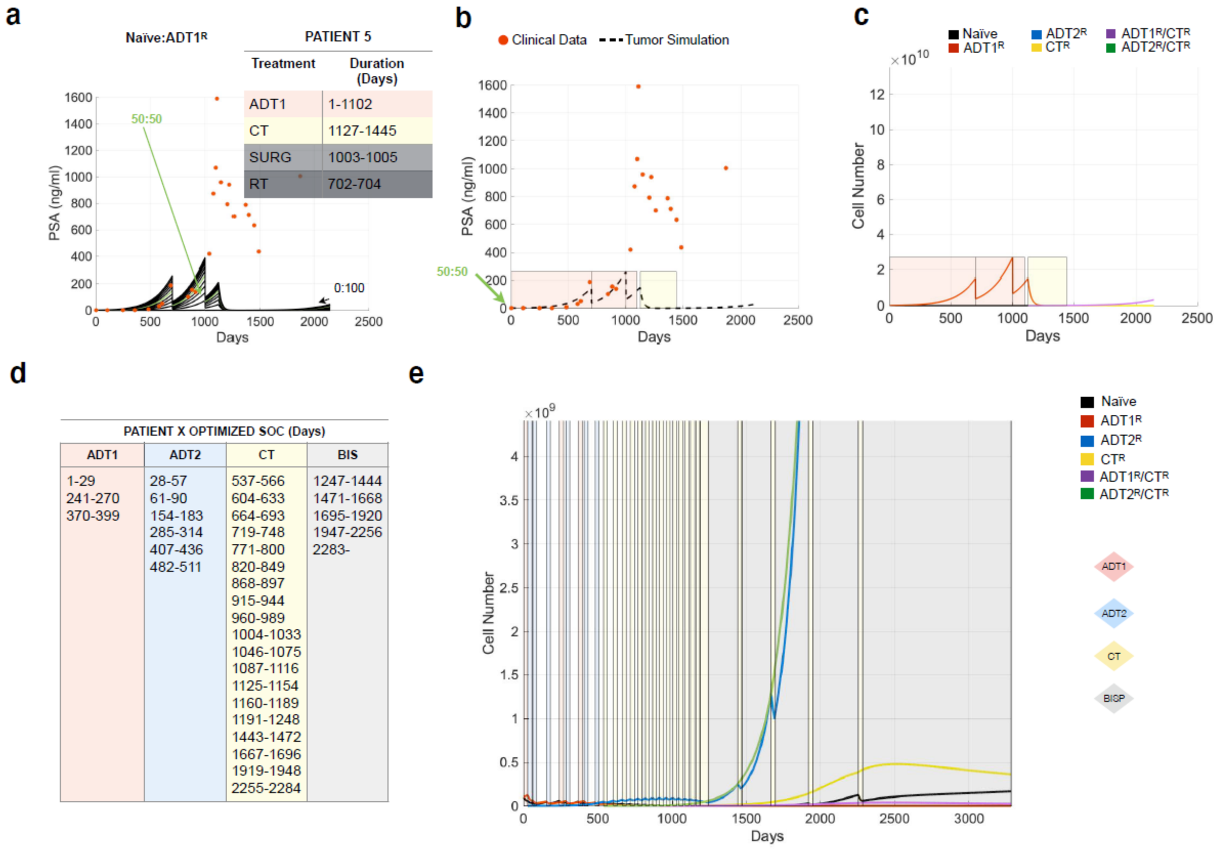

PATIENT X OPTIMIZED SOC (Days)

ADT1	ADT2	CT	BIS
1-29	28-57	537-566	1247-1444
241-270	61-90	604-633	1471-1668
370-399	154-183	664-693	1695-1920
	285-314	719-748	1947-2256
	407-436	771-800	2283-
	482-511	820-849	
		868-897	
		915-944	
		960-989	
		1004-1033	
		1046-1075	
		1087-1116	
		1125-1154	
		1160-1189	
		1191-1248	
		1443-1472	
		1667-1696	
		1919-1948	
		2255-2284	

Figure S6. Araujo et al.

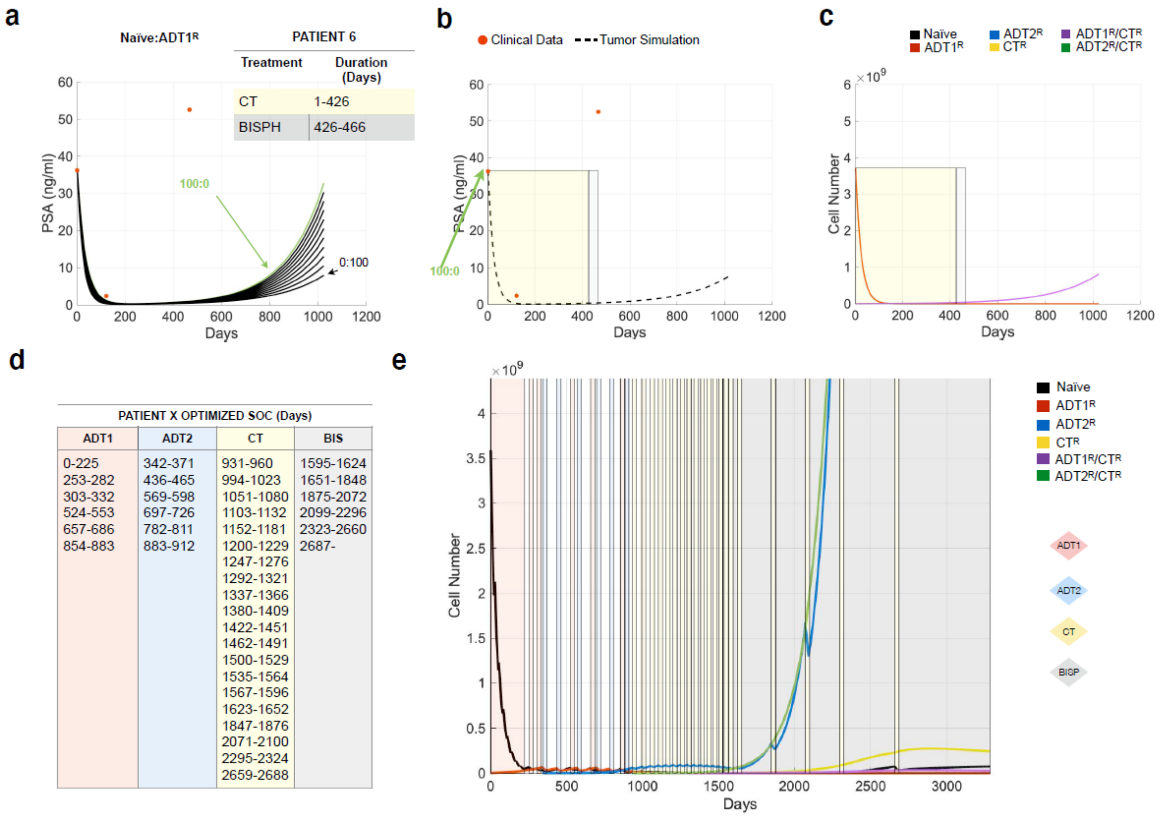

PATIENT X OPTIMIZED SOC (Days)

ADT1	ADT2	CT	BIS
0-225	342-371	931-960	1595-1624
253-282	436-465	994-1023	1651-1848
303-332	569-598	1051-1080	1875-2072
524-553	697-726	1103-1132	2099-2296
657-686	782-811	1152-1181	2323-2660
854-883	883-912	1200-1229	2687-
		1247-1276	
		1292-1321	
		1337-1366	
		1380-1409	
		1422-1451	
		1462-1491	
		1500-1529	
		1535-1564	
		1567-1596	
		1623-1652	
		1847-1876	
		2071-2100	
		2295-2324	
		2659-2688	

Figure S7. Araujo et al.

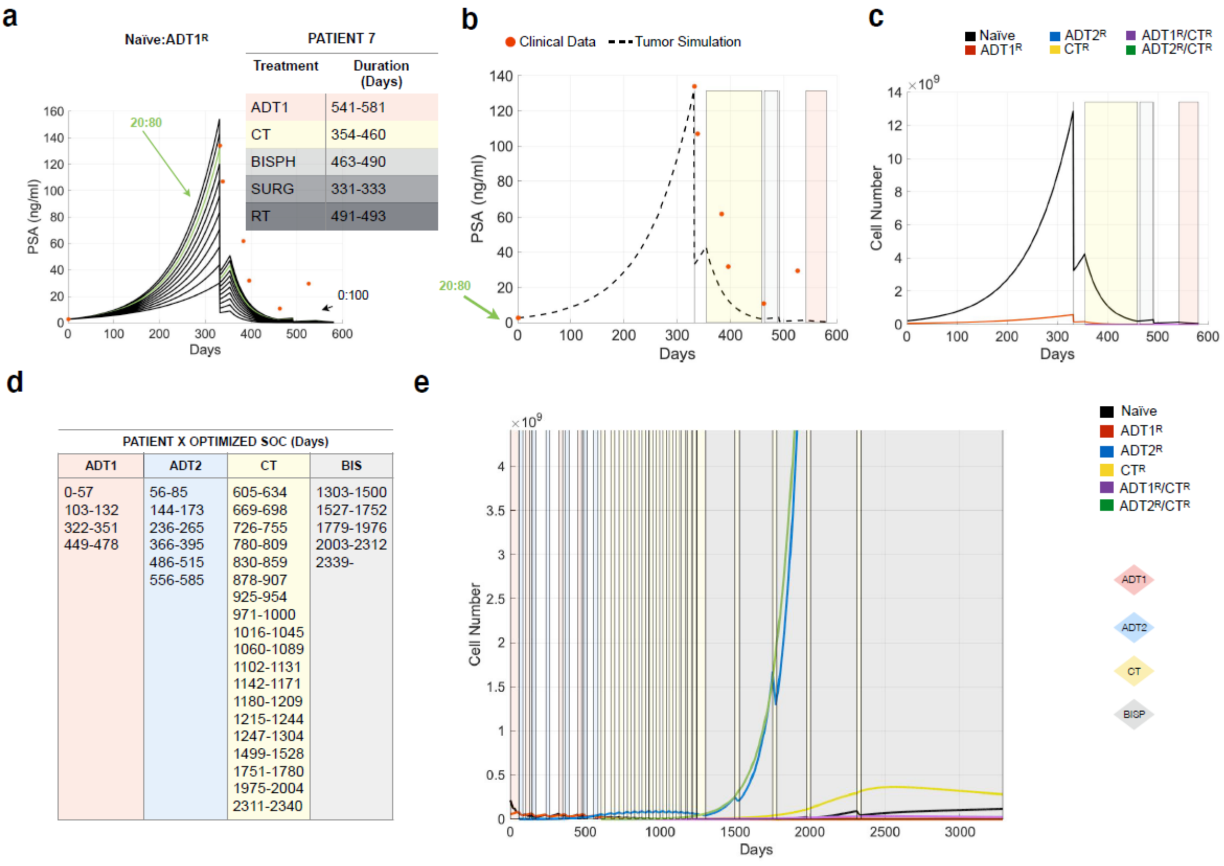

PATIENT 7	
Treatment	Duration (Days)
ADT1	541-581
CT	354-460
BISPH	463-490
SURG	331-333
RT	491-493

PATIENT X OPTIMIZED SOC (Days)

ADT1	ADT2	CT	BIS
0-57	56-85	605-634	1303-1500
103-132	144-173	669-698	1527-1752
322-351	236-265	726-755	1779-1976
449-478	366-395	780-809	2003-2312
	486-515	830-859	2339-
	556-585	878-907	
		925-954	
		971-1000	
		1016-1045	
		1060-1089	
		1102-1131	
		1142-1171	
		1180-1209	
		1215-1244	
		1247-1304	
		1499-1528	
		1751-1780	
		1975-2004	
		2311-2340	

Figure S8. Araujo et al.

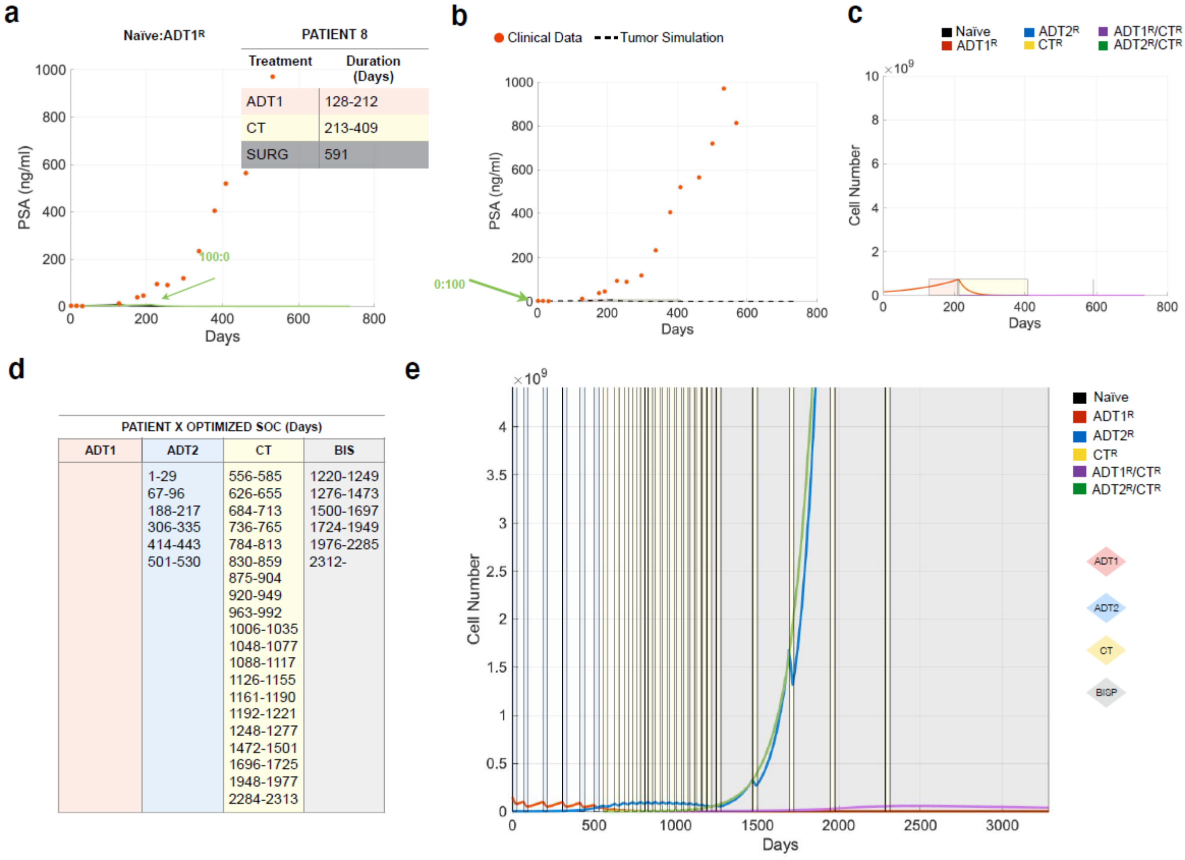

PATIENT 8	
Treatment	Duration (Days)
ADT1	128-212
CT	213-409
SURG	591

PATIENT X OPTIMIZED SOC (Days)

ADT1	ADT2	CT	BIS
	1-29	556-585	1220-1249
	67-96	626-655	1276-1473
	188-217	684-713	1500-1697
	306-335	736-765	1724-1949
	414-443	784-813	1976-2285
	501-530	830-859	2312-
		875-904	
		920-949	
		963-992	
		1006-1035	
		1048-1077	
		1088-1117	
		1126-1155	
		1161-1190	
		1192-1221	
		1248-1277	
		1472-1501	
		1696-1725	
		1948-1977	
		2284-2313	

Figure S9. Araujo et al.

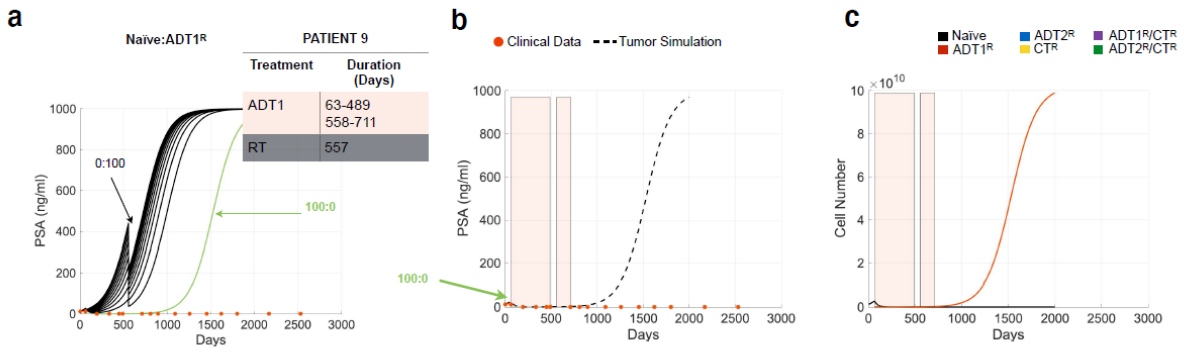

d

PATIENT X OPTIMIZED SOC (Days)			
ADT1	ADT2	CT	BIS
1-141	449-478	1020-1049	1740-1937
149-178	583-612	1092-1121	1964-2161
217-246	714-743	1155-1184	2188-2413
282-311	840-869	1212-1241	2440-2721
344-373	924-953	1264-1293	2748-
401-430		1314-1343	
537-566		1362-1391	
672-701		1408-1437	
803-832		1453-1482	
992-1021		1497-1526	
		1539-1568	
		1580-1609	
		1618-1647	
		1653-1682	
		1684-1741	
		1936-1965	
		2160-2189	
		2412-2441	
		2720-2749	

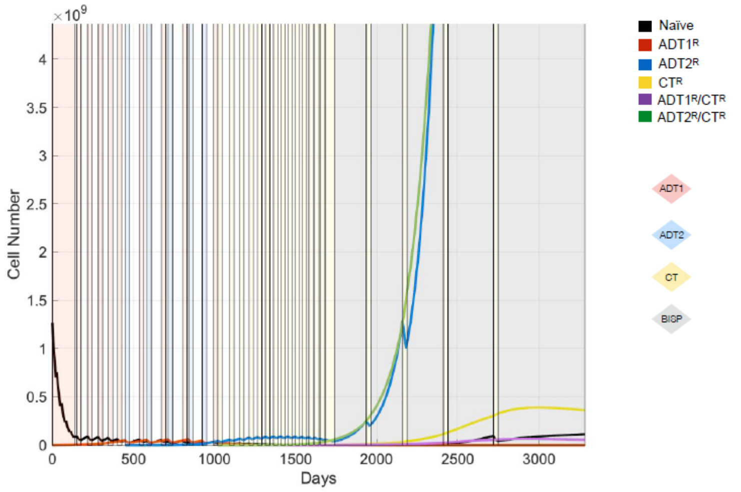

Figure S10. Araujo et al.

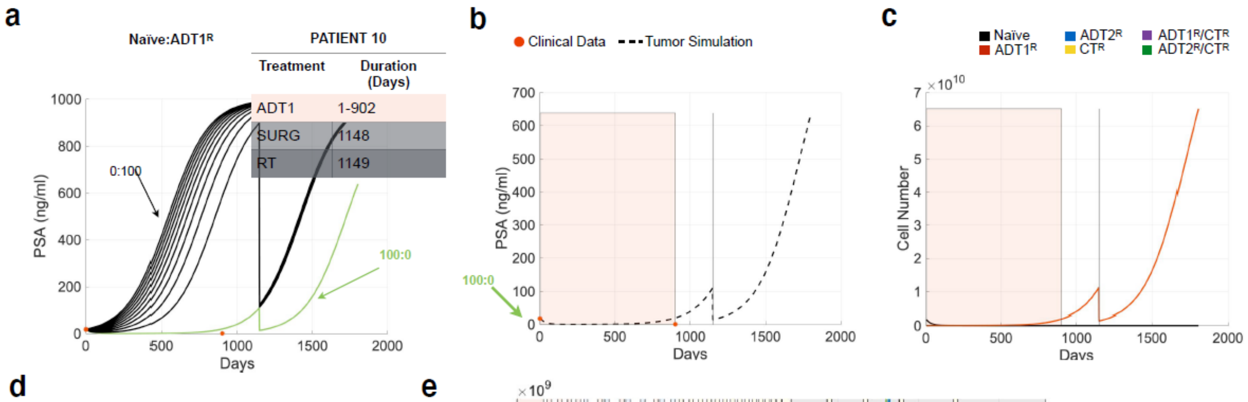

d

PATIENT X OPTIMIZED SOC (Days)			
ADT1	ADT2	CT	BIS
1-169	415-444	1008-1037	1705-1930
189-218	547-576	1071-1100	1957-2154
254-283	678-707	1129-1158	2181-2378
315-344	769-798	1182-1211	2405-2714
369-398	889-918	1232-1261	2741-
505-534	958-987	1281-1310	
639-668		1328-1357	
851-880		1374-1403	
		1418-1447	
		1462-1491	
		1504-1533	
		1545-1574	
		1583-1612	
		1618-1647	
		1649-1706	
		1929-1958	
		2153-2182	
		2377-2406	
		2713-2742	

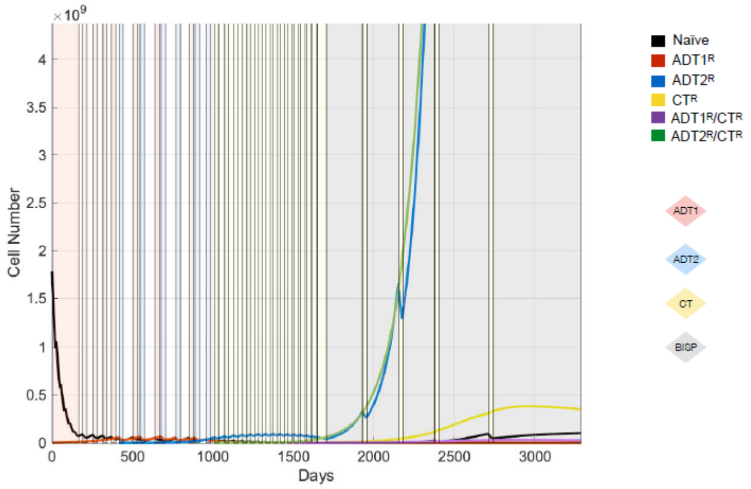

Figure S11. Araujo et al.

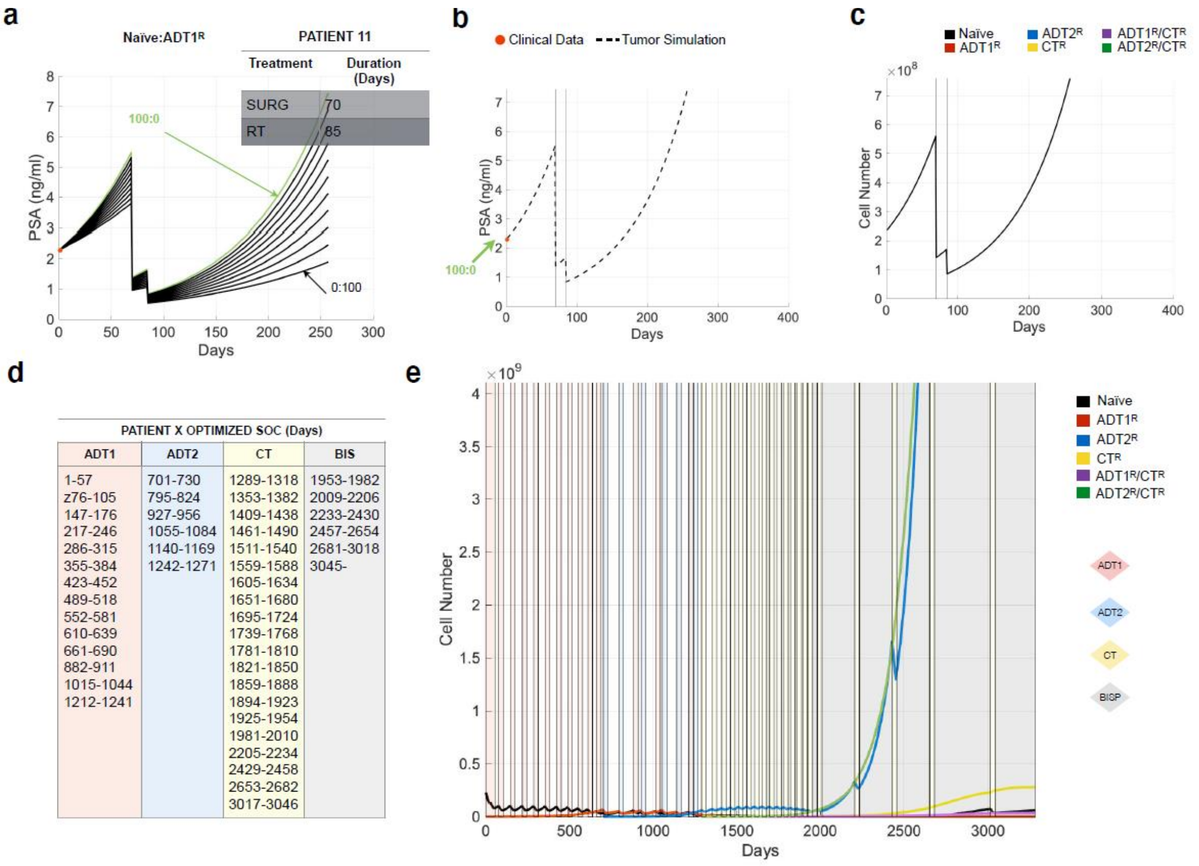

PATIENT X OPTIMIZED SOC (Days)

ADT1	ADT2	CT	BIS
1-57	701-730	1289-1318	1953-1982
z76-105	795-824	1353-1382	2009-2206
147-176	927-956	1409-1438	2233-2430
217-246	1055-1084	1461-1490	2457-2654
286-315	1140-1169	1511-1540	2681-3018
355-384	1242-1271	1559-1588	3045-
423-452		1605-1634	
489-518		1651-1680	
552-581		1695-1724	
610-639		1739-1768	
661-690		1781-1810	
882-911		1821-1850	
1015-1044		1859-1888	
1212-1241		1894-1923	
		1925-1954	
		1981-2010	
		2205-2234	
		2429-2458	
		2653-2682	
		3017-3046	

Figure S12. Araujo et al.

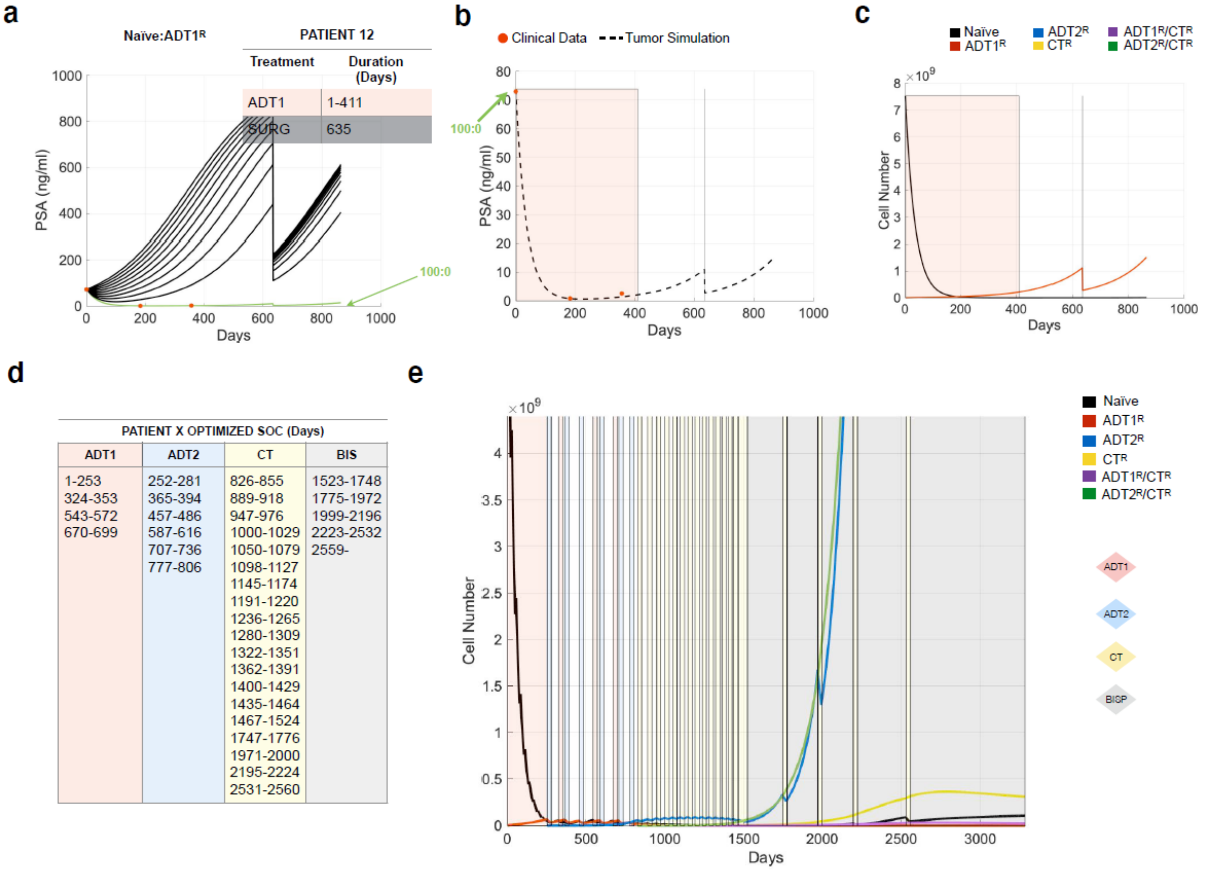

PATIENT X OPTIMIZED SOC (Days)

ADT1	ADT2	CT	BIS
1-253	252-281	826-855	1523-1748
324-353	365-394	889-918	1775-1972
543-572	457-486	947-976	1999-2196
670-699	587-616	1000-1029	2223-2532
	707-736	1050-1079	2559-
	777-806	1098-1127	
		1145-1174	
		1191-1220	
		1236-1265	
		1280-1309	
		1322-1351	
		1362-1391	
		1400-1429	
		1435-1464	
		1467-1524	
		1747-1776	
		1971-2000	
		2195-2224	
		2531-2560	

Figure S13. Araujo et al.

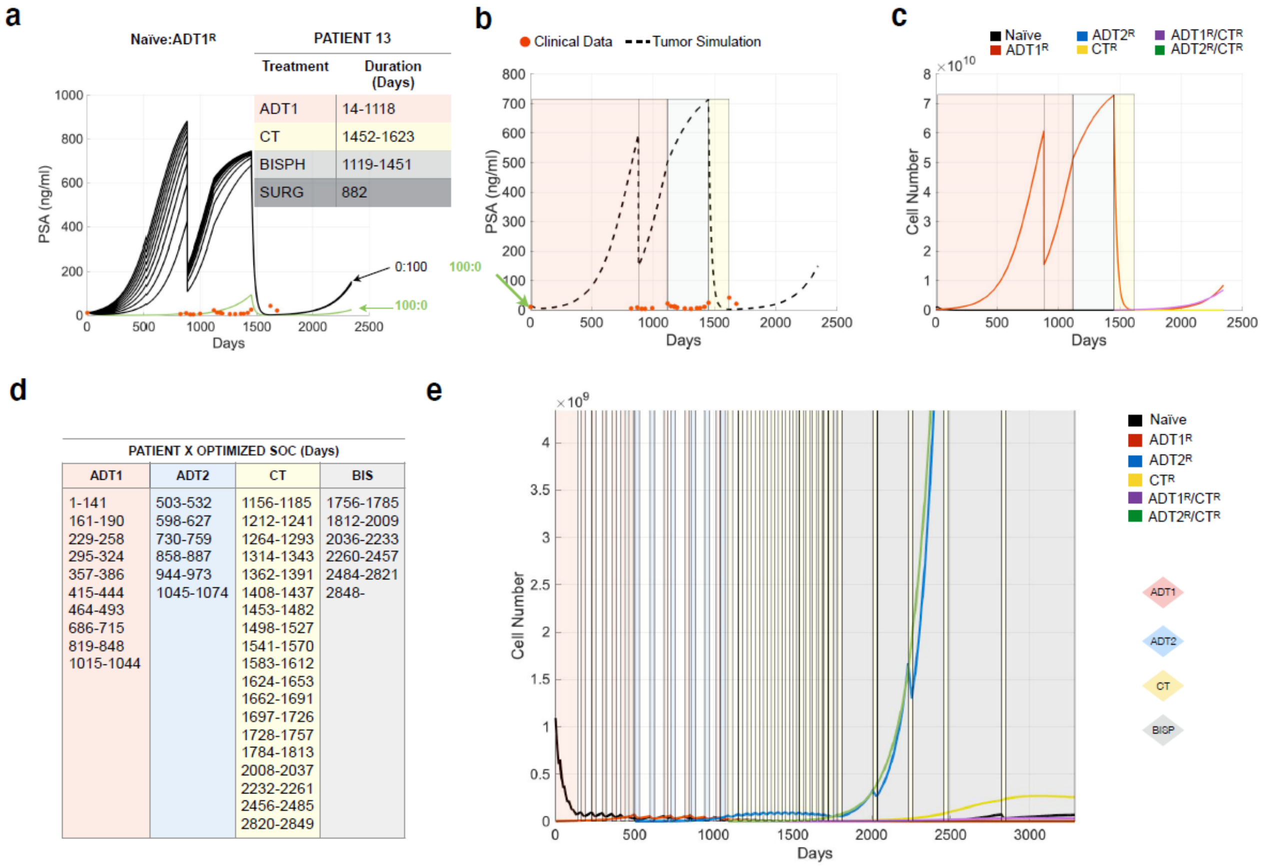

Figure S14. Araujo et al.

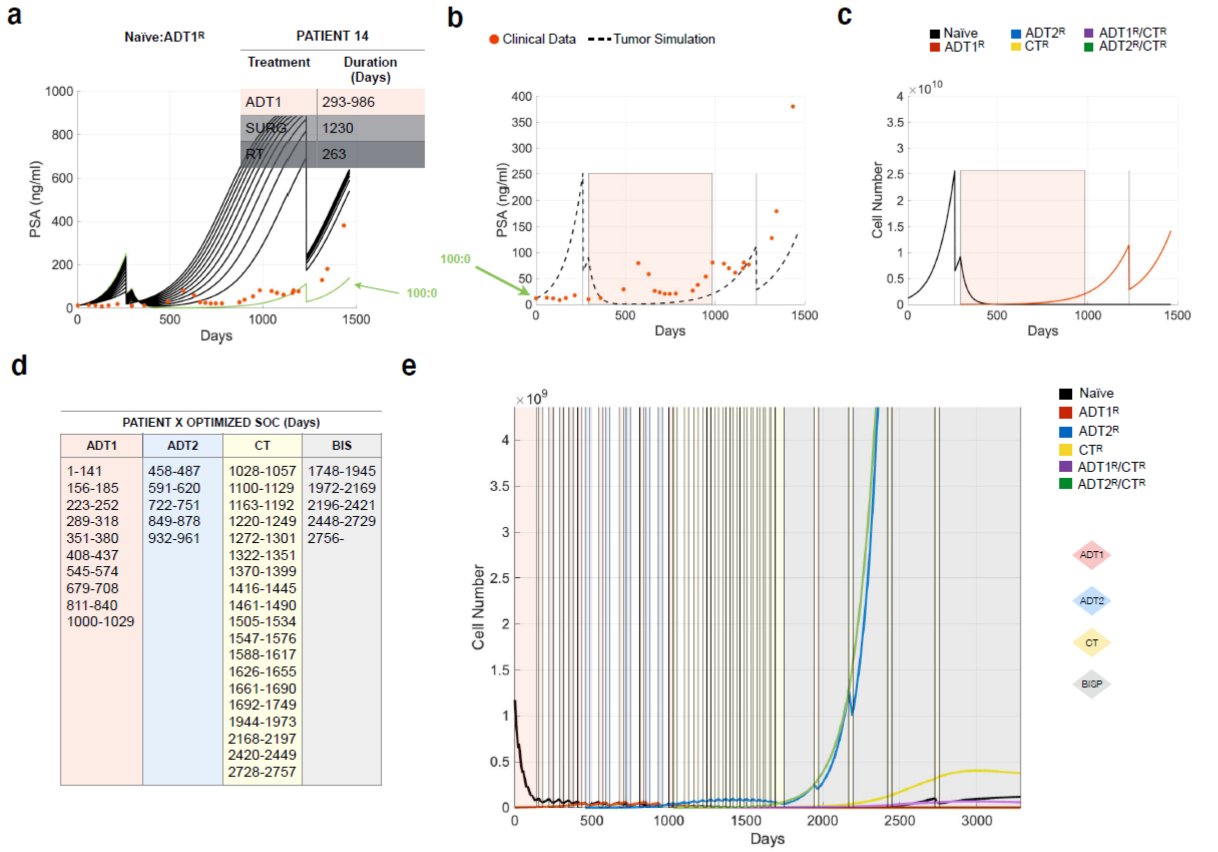

Figure S15. Araujo et al.

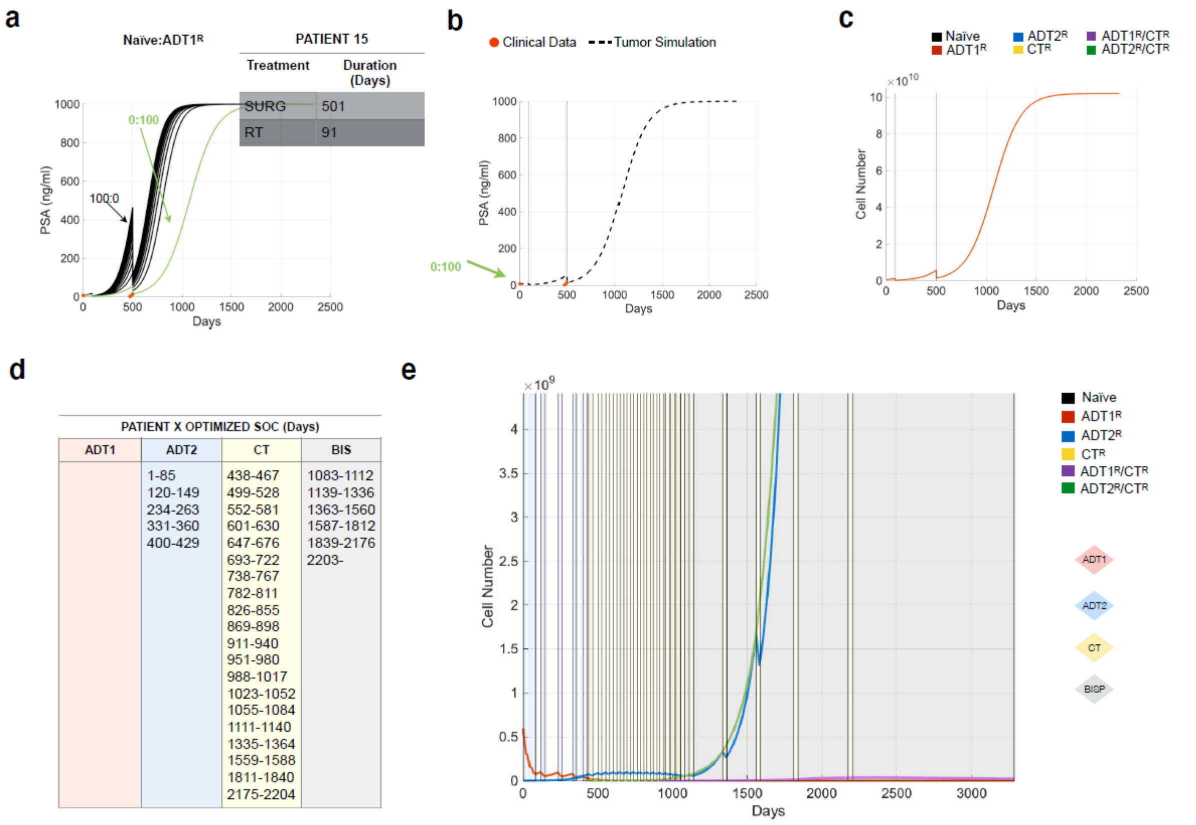

Figure S16. Araujo et al.

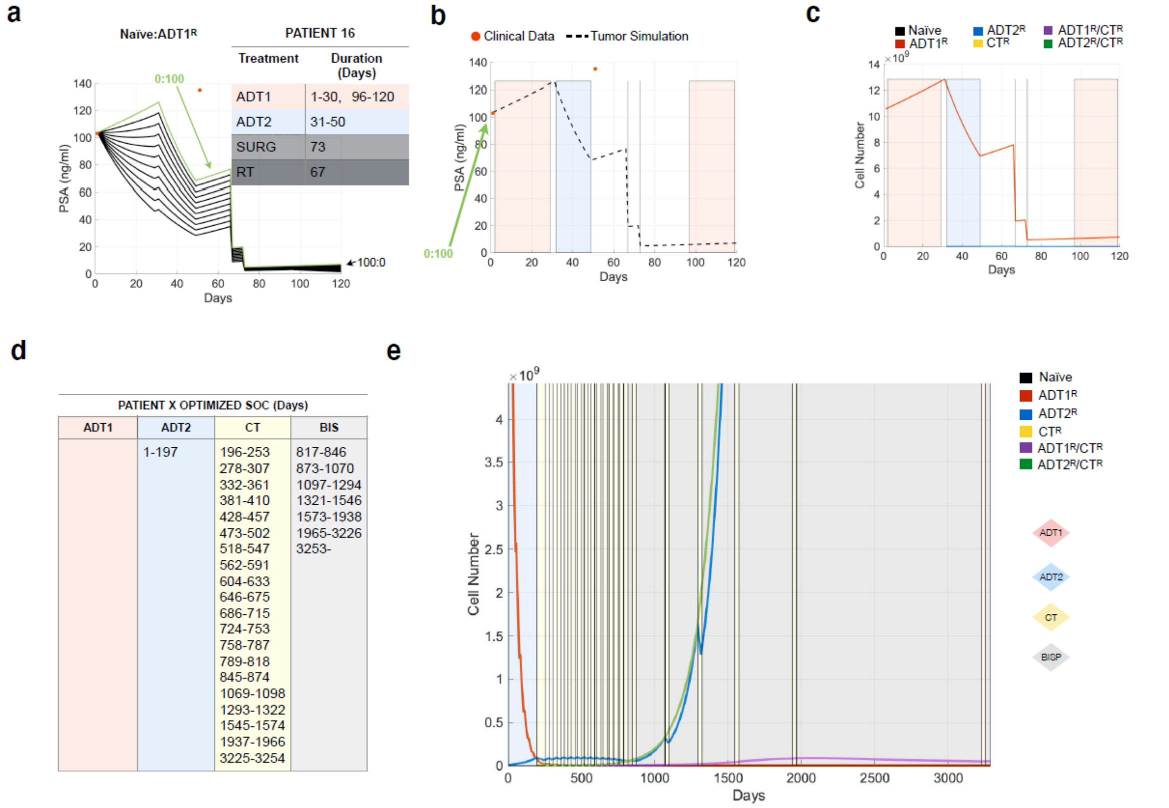

Figure S17. Araujo et al.

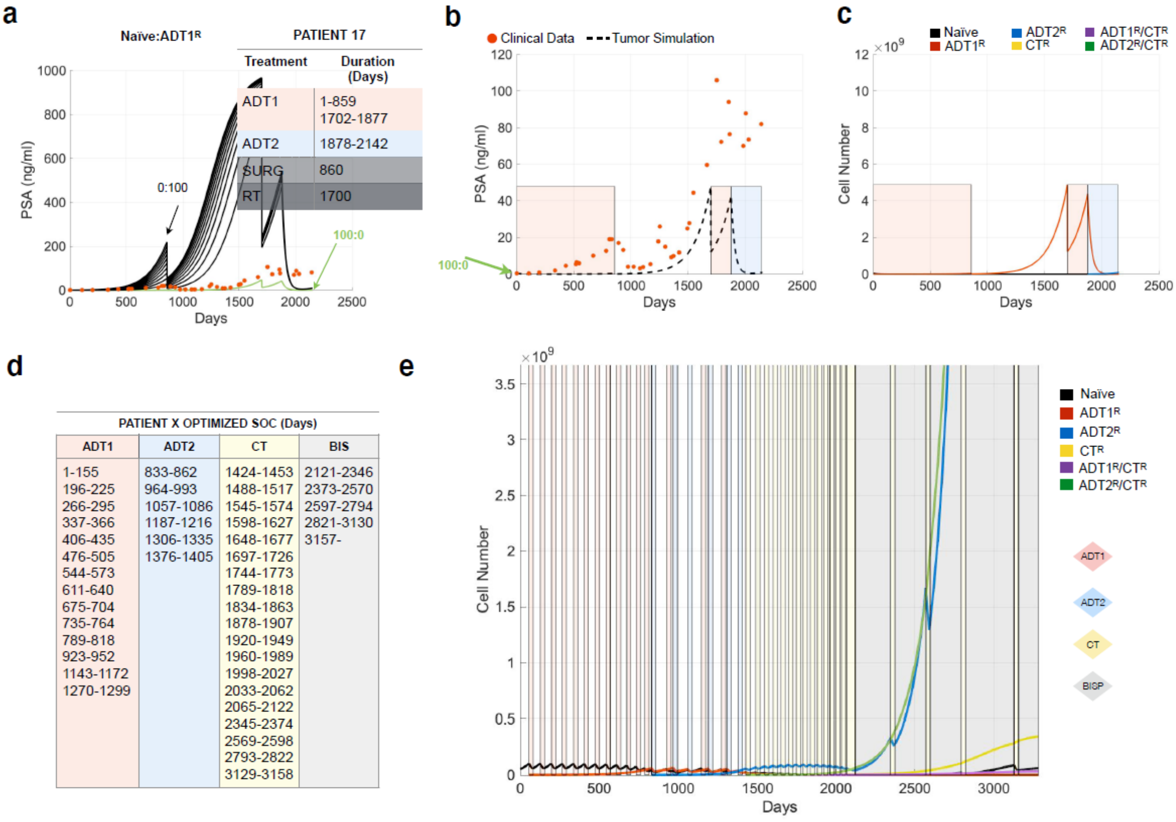

Figure S18. Araujo et al.

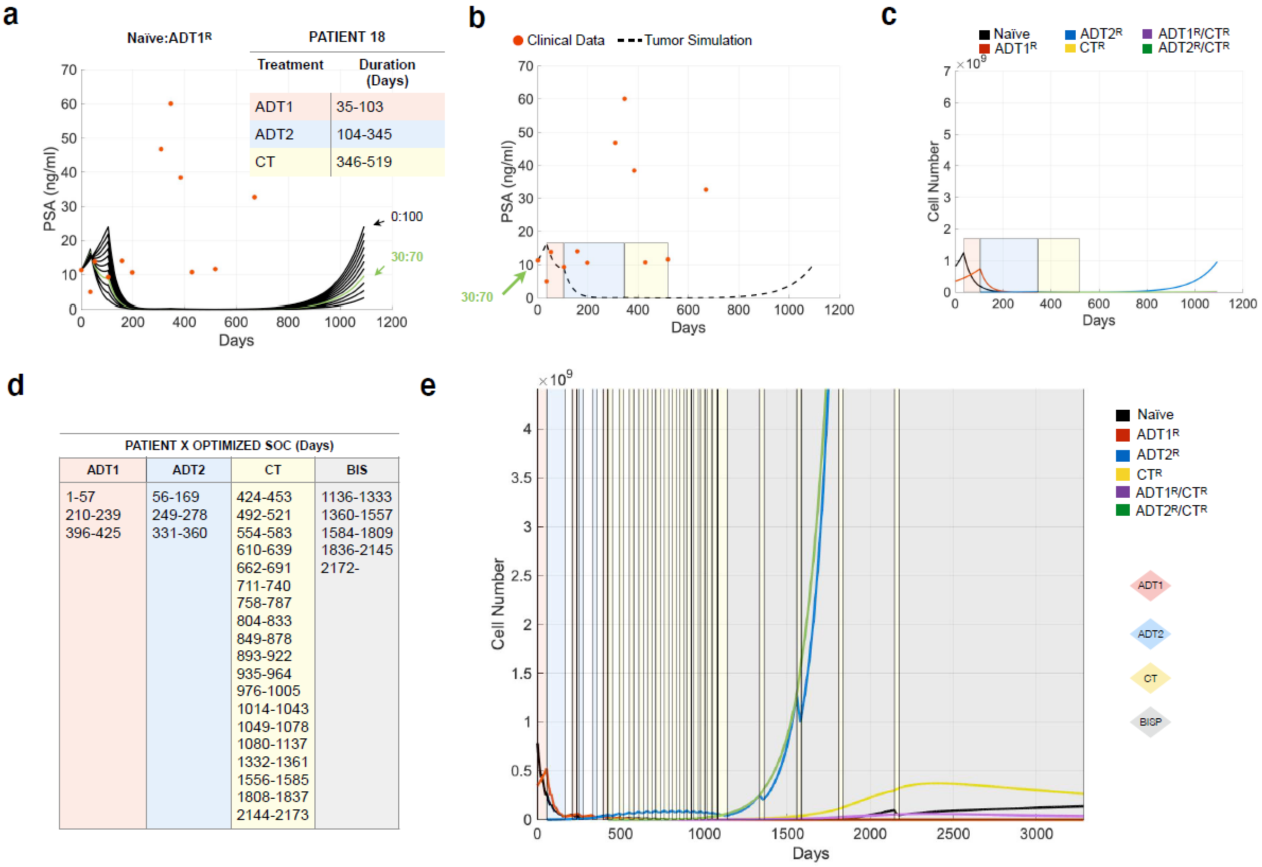

Figure S19. Araujo et al.

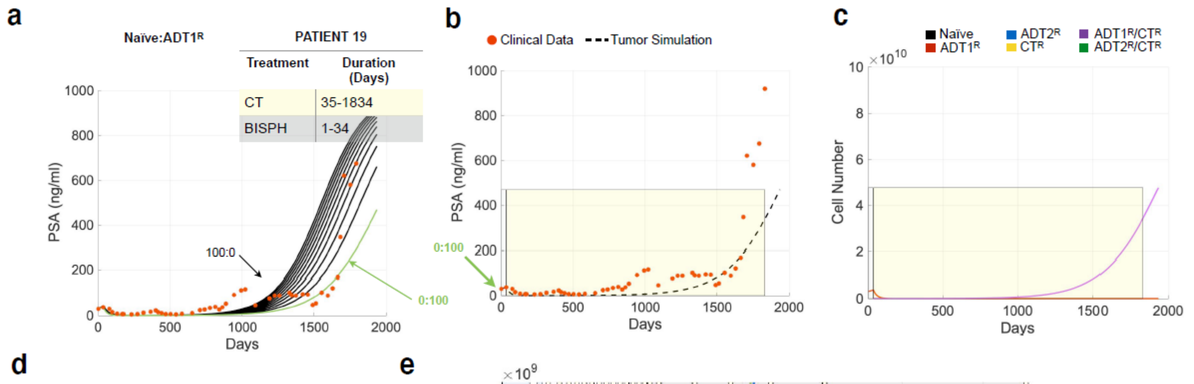

d

PATIENT X OPTIMIZED SOC (Days)			
ADT1	ADT2	CT	BIS
	1-169	269-298	932-961
	215-244	339-368	988-1185
		396-425	1212-1409
		447-476	1436-1661
		495-524	1688-1997
		541-570	2024-3257
		587-616	3284-
		631-660	
		675-704	
		718-747	
		759-788	
		799-828	
		837-866	
		872-901	
		904-933	
		960-989	
		1184-1213	
		1408-1437	
		1660-1689	
		1996-2025	
		3256-3285	

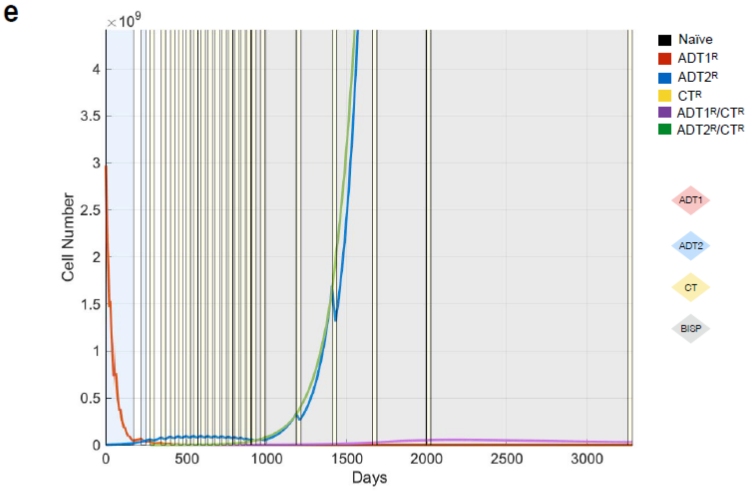

Figure S20. Araujo et al.

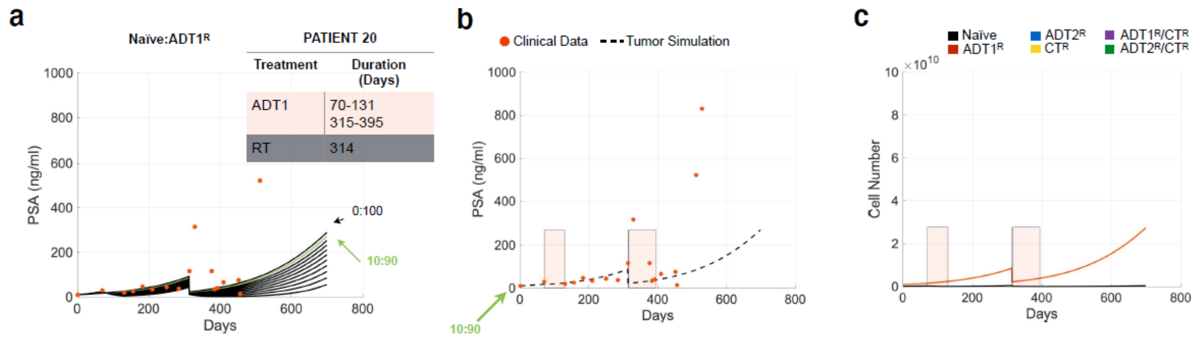

d

PATIENT X OPTIMIZED SOC (Days)			
ADT1	ADT2	CT	BIS
	1-113	365-394	1032-1061
	152-181	430-459	1088-1285
	243-272	487-516	1312-1509
	316-345	539-568	1536-1733
		589-618	1760-2097
		637-666	2124-
		684-713	
		729-758	
		774-803	
		817-846	
		859-888	
		899-928	
		937-966	
		973-1002	
		1004-1033	
		1060-1089	
		1284-1313	
		1508-1537	
		1732-1761	
		2096-2125	

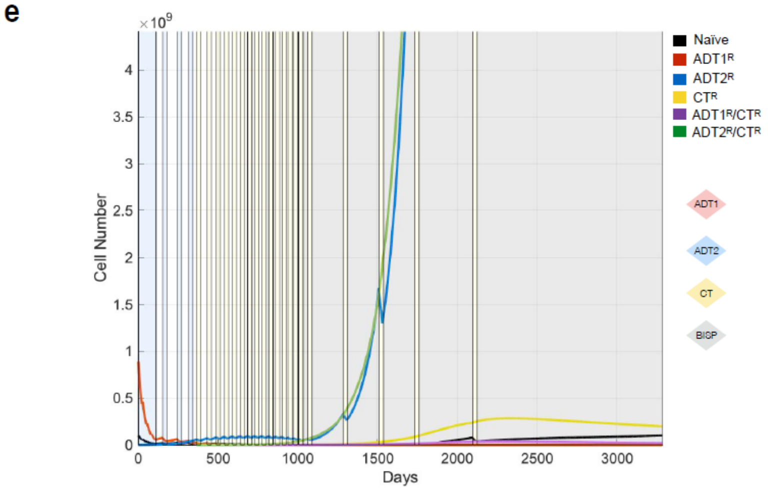

Figure S21. Araujo et al.

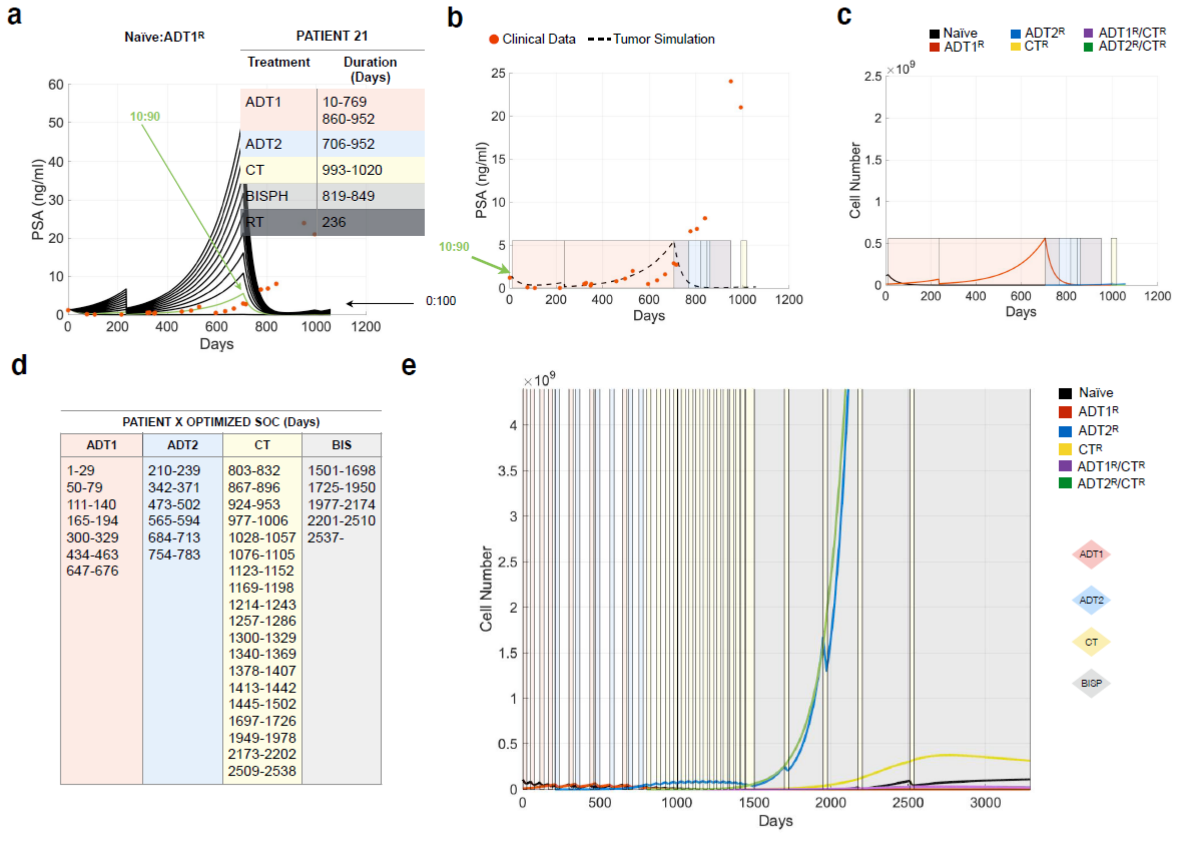

Treatment	Duration (Days)
ADT1	10-769<br>860-952
ADT2	706-952
CT	993-1020
BISPH	819-849
RT	236

PATIENT X OPTIMIZED SOC (Days)

ADT1	ADT2	CT	BIS
1-29	210-239	803-832	1501-1698
50-79	342-371	867-896	1725-1950
111-140	473-502	924-953	1977-2174
165-194	565-594	977-1006	2201-2510
300-329	684-713	1028-1057	2537-
434-463	754-783	1076-1105	
647-676		1123-1152	
		1169-1198	
		1214-1243	
		1257-1286	
		1300-1329	
		1340-1369	
		1378-1407	
		1413-1442	
		1445-1502	
		1697-1726	
		1949-1978	
		2173-2202	
		2509-2538	

Figure S22. Araujo et al.

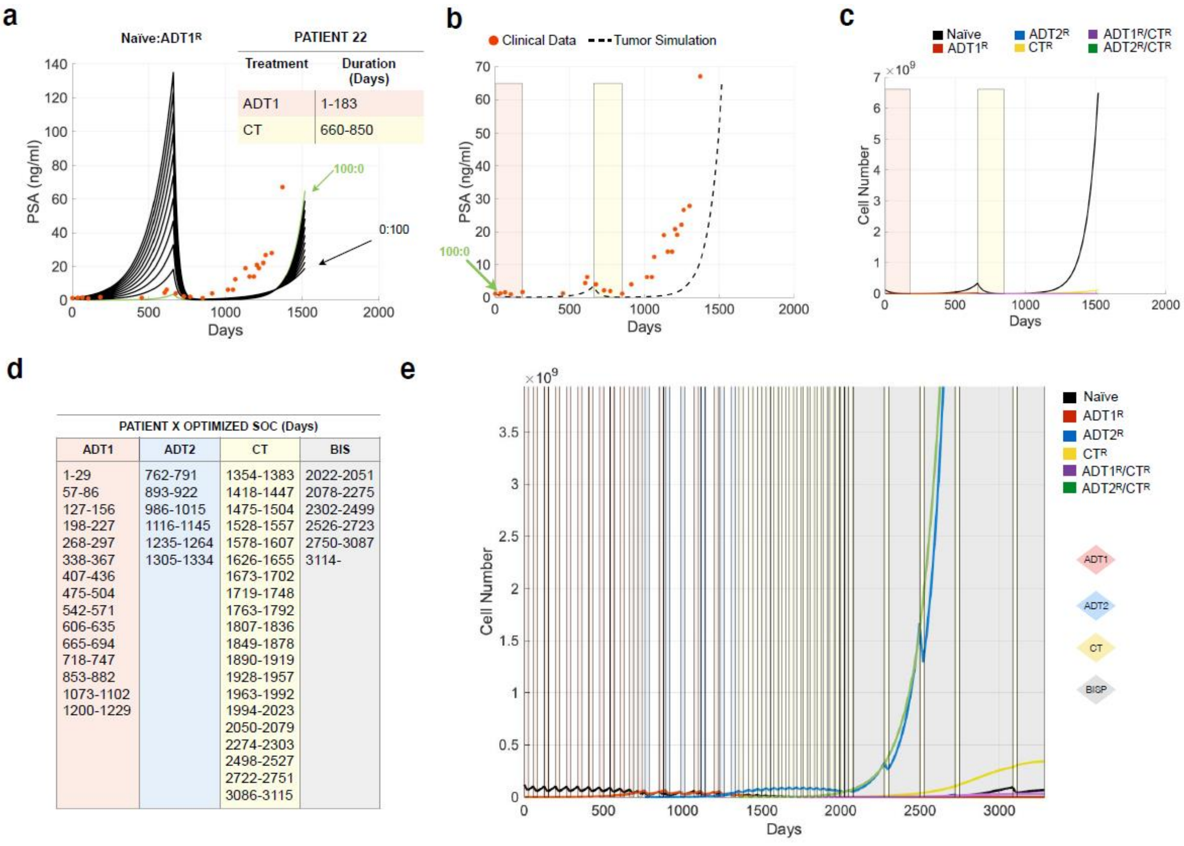

Treatment	Duration (Days)
ADT1	1-183
CT	660-850

PATIENT X OPTIMIZED SOC (Days)

ADT1	ADT2	CT	BIS
1-29	762-791	1354-1383	2022-2051
57-86	893-922	1418-1447	2078-2275
127-156	986-1015	1475-1504	2302-2499
198-227	1116-1145	1528-1557	2526-2723
268-297	1235-1264	1578-1607	2750-3087
338-367	1305-1334	1626-1655	3114-
407-436		1673-1702	
475-504		1719-1748	
542-571		1763-1792	
606-635		1807-1836	
665-694		1849-1878	
718-747		1890-1919	
853-882		1928-1957	
1073-1102		1963-1992	
1200-1229		1994-2023	
		2050-2079	
		2274-2303	
		2498-2527	
		2722-2751	
		3086-3115	

Figure S23. Araujo et al.

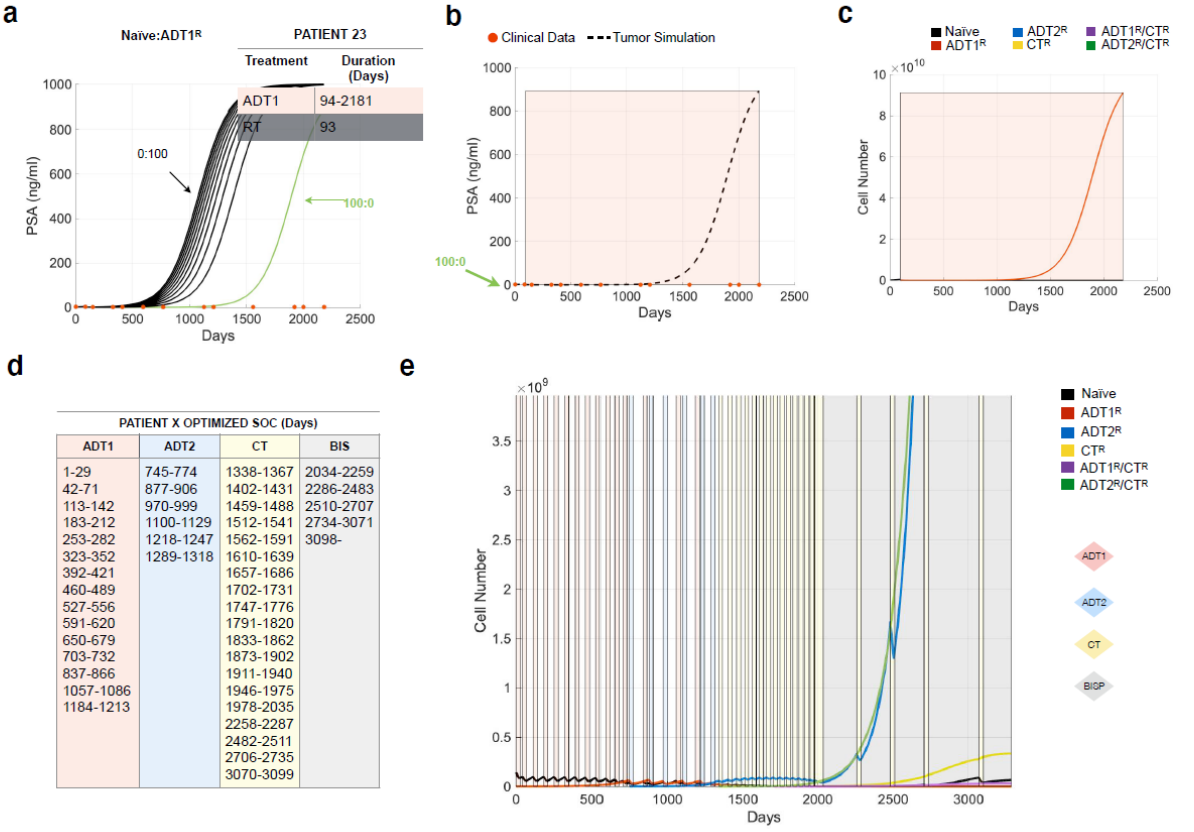

ADT1	ADT2	CT	BIS
1-29	745-774	1338-1367	2034-2259
42-71	877-906	1402-1431	2286-2483
113-142	970-999	1459-1488	2510-2707
183-212	1100-1129	1512-1541	2734-3071
253-282	1218-1247	1562-1591	3098-
323-352	1289-1318	1610-1639	
392-421		1657-1686	
460-489		1702-1731	
527-556		1747-1776	
591-620		1791-1820	
650-679		1833-1862	
703-732		1873-1902	
837-866		1911-1940	
1057-1086		1946-1975	
1184-1213		1978-2035	
		2258-2287	
		2482-2511	
		2706-2735	
		3070-3099	

Figure S24. Araujo et al.

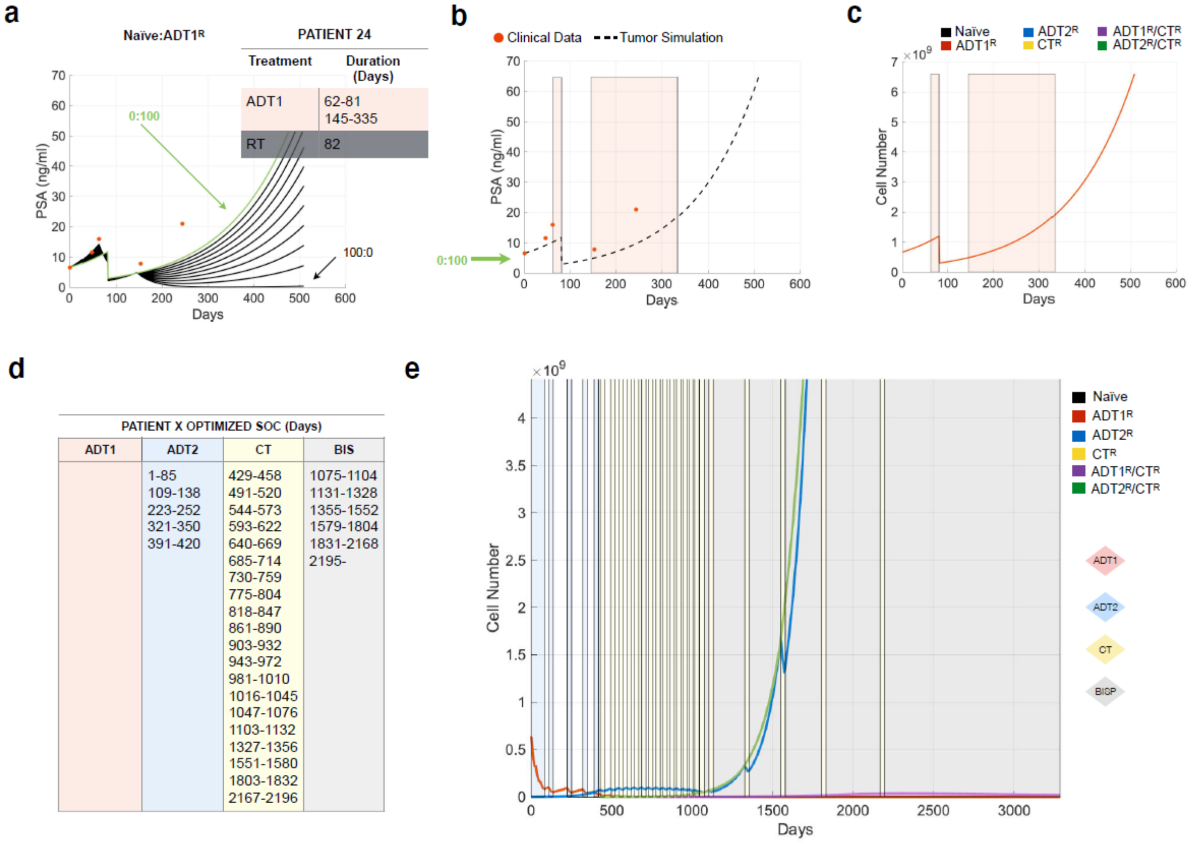

ADT1	ADT2	CT	BIS
	1-85	429-458	1075-1104
	109-138	491-520	1131-1328
	223-252	544-573	1355-1552
	321-350	593-622	1579-1804
	391-420	640-669	1831-2168
		685-714	2195-
		730-759	
		775-804	
		818-847	
		861-890	
		903-932	
		943-972	
		981-1010	
		1016-1045	
		1047-1076	
		1103-1132	
		1327-1356	
		1551-1580	
		1803-1832	
		2167-2196	

Figure S25. Araujo et al.
